# Clonal evolution in patients with chronic lymphocytic leukaemia developing resistance to BTK inhibition

**DOI:** 10.1038/ncomms11589

**Published:** 2016-05-20

**Authors:** Jan A. Burger, Dan A. Landau, Amaro Taylor-Weiner, Ivana Bozic, Huidan Zhang, Kristopher Sarosiek, Lili Wang, Chip Stewart, Jean Fan, Julia Hoellenriegel, Mariela Sivina, Adrian M. Dubuc, Cameron Fraser, Yulong Han, Shuqiang Li, Kenneth J. Livak, Lihua Zou, Youzhong Wan, Sergej Konoplev, Carrie Sougnez, Jennifer R. Brown, Lynne V. Abruzzo, Scott L. Carter, Michael J. Keating, Matthew S. Davids, William G. Wierda, Kristian Cibulskis, Thorsten Zenz, Lillian Werner, Paola Dal Cin, Peter Kharchencko, Donna Neuberg, Hagop Kantarjian, Eric Lander, Stacey Gabriel, Susan O'Brien, Anthony Letai, David A. Weitz, Martin A. Nowak, Gad Getz, Catherine J. Wu

**Affiliations:** 1 Department of Leukemia, MD Anderson Cancer Center, Houston, Texas 77030, USA; 2 Broad Institute, Cambridge, Massachusetts 02142, USA; 3 Department of Medicine, Weill Cornell Medicine, New York, New York 10065, USA; 4 Department of Physiology and Biophysics, Weill Cornell Medicine, New York, New York 10065, USA; 5 New York Genome Center, New York, New York 10013, USA; 6 Department of Mathematics, Program for Evolutionary Dynamics, Harvard University, Cambridge, Massachusetts 02138, USA; 7 Department of Organismic and Evolutionary Biology, Harvard University, Cambridge, Massachusetts 02138, USA; 8 Department of Physics, School of Engineering and Applied Sciences, Harvard University, Cambridge, Massachusetts 02138, USA; 9 Department of Cell Biology, Key Laboratory of Cell Biology, Ministry of Public Health, China Medical University, Shenyang 110001, China; 10 Key Laboratory of Medical Cell Biology, Ministry of Education, China Medical University, Shenyang 110001, China; 11 Department of Medical Oncology, Dana-Farber Cancer Institute, Boston, Massachusetts 02215, USA; 12 Center for Biomedical Informatics, Harvard Medical School, Boston Massachusetts 02115, USA; 13 Department of Pathology, Brigham and Women's Hospital and Harvard Medical School, Boston, Massachusetts 02115, USA; 14 Bioinspired Engineering and Biomechanics Center, Xi'an Jiaotong University, Xi'an 710049, China; 15 Fluidigm Corporation, South San Francisco, California 94080, USA; 16 Department of Hematopathology, MD Anderson Cancer Center, Houston, Texas 77030, USA; 17 National Center for Tumors and German Cancer Research Center (DKFZ), 69121 Heidelberg, Germany; 18 Biostatistics and Computational Biology, Dana-Farber Cancer Institute, Boston, Massachusetts, USA; 19 Department of Medicine, Brigham & Women's Hospital, Harvard Medical School, Boston, Massachusetts 02215, USA

## Abstract

Resistance to the Bruton's tyrosine kinase (BTK) inhibitor ibrutinib has been attributed solely to mutations in *BTK* and related pathway molecules. Using whole-exome and deep-targeted sequencing, we dissect evolution of ibrutinib resistance in serial samples from five chronic lymphocytic leukaemia patients. In two patients, we detect *BTK-C481S* mutation or multiple *PLCG2* mutations. The other three patients exhibit an expansion of clones harbouring *del*(8p) with additional driver mutations (*EP300, MLL2* and *EIF2A*), with one patient developing *trans*-differentiation into CD19-negative histiocytic sarcoma. Using droplet-microfluidic technology and growth kinetic analyses, we demonstrate the presence of ibrutinib-resistant subclones and estimate subclone size before treatment initiation. Haploinsufficiency of TRAIL-R, a consequence of *del*(8p), results in TRAIL insensitivity, which may contribute to ibrutinib resistance. These findings demonstrate that the ibrutinib therapy favours selection and expansion of rare subclones already present before ibrutinib treatment, and provide insight into the heterogeneity of genetic changes associated with ibrutinib resistance.

## Abstract (web summary)

The BTK inhibitor ibrutinib is used to treat chronic lymphocytic leukaemia, however some patients develop resistance to the drug. Here, the authors use genomic analyses to examine the clonal evolution of 5 patients that develop resistance to ibrutinib.

## Graphical abstract

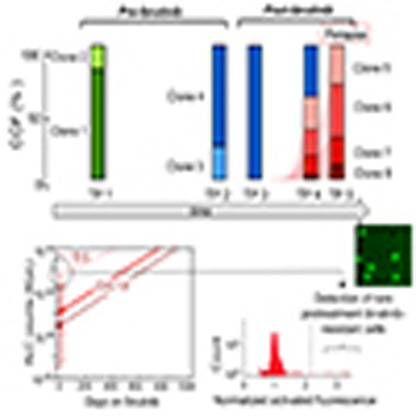

B-cell receptor (BCR) signalling is a critical growth and survival pathway in several B-cell malignancies, including chronic lymphocytic leukaemia (CLL)^1^. BCR signalling can be abrogated by novel kinase inhibitors that target the BCR-associated kinases SYK^2^, Bruton's tyrosine kinase (BTK)^3^ and PI3Kδ (ref. 4). The BTK inhibitor ibrutinib is a small molecule that inactivates BTK through irreversible covalent binding to Cys-481 within the ATP-binding domain of BTK^5^. In a recent trial in patients with relapsed/refractory CLL, ibrutinib induced an overall response rate of 71% and an estimated progression-free survival rate of 75% after 26 months of therapy^3^. However, a small fraction of patients develop progressive CLL after initially responding to ibrutinib^3^. Among these, patients carrying *BTK* mutations at the ibrutinib-binding site (C481S) or affecting the BCR-signalling-related molecule *PLCγ2* (R665W, L845F and S707Y) were recently highlighted^6^,^7^.

The ability of cancer cells to evolve and adapt to targeted therapies is a challenge that limits treatment success and durability of responses. Whole-exome sequencing (WES), along with analyses of clonal heterogeneity and clonal evolution in CLL, can provide insight into emergence and expansion of subclones that carry driver mutations (for example, *SF3B1* and *TP53*) under therapeutic pressure^8^. To determine the pattern of clonal evolution in ibrutinib-resistant CLL patients, we performed a longitudinal genomic investigation of five CLL patients who achieved partial remissions and later experienced disease progression. Here we demonstrate that ibrutinib therapy favours the selection and expansion of CLL subclones, carrying *del*(8p) with additional driver mutations in three different patients. Our studies provide insight into dynamic changes in clonal architecture during ibrutinib therapy, as well as potential alternative mechanisms of resistance. These findings are of particular interest, given the increasing clinical use of this BTK inhibitor.

## Results

### CLL relapse following ibrutinib therapy in Patients 1–4

Ibrutinib therapy generally is highly effective, and responses are durable in the vast majority of patients. However, some patients who initially respond the CLL eventually relapse because of the development of resistance, and presence of *del*(17p), especially in combination with other complex cytogenetic alterations, has been reported to predispose CLL patients for development of resistance^9^. In patients with relapsed/refractory CLL, the reported rate of ibrutinib resistance at 26 months was 13% (ref. 3) or 7.5% at a median follow-up of 16.8 months (ref. 10). Five patients from these early clinical trials were characterized in this report. At ibrutinib treatment initiation, Patients 1–4 had advanced stage CLL (Rai stage 3–4; Table 1). Patients 1 and 2 had relapsed disease after fludarabine, cyclophosphamide and rituximab (FCR) frontline therapy, while Patients 3 and 4 had received multiple lines of prior therapy. CLL samples from all patients harboured high-risk cytogenetic abnormalities (Patients 1 and 3 with *del*(17p) and Patients 2 and 4 with complex cytogenetic including *del*(11q) and *del*(17p). As the best response to ibrutinib-based therapy, all four experienced partial remissions. Patient 1 demonstrated normalization of haematologic parameters after 183 days, with persistent bone marrow disease (12% residual CLL cells after 448 days on ibrutinib). Patient 2 had normalization of haematologic parameters after 87 days with resolution of lymphadenopathy and splenomegaly, but persistent marrow disease (29% residual CLL cells). Patient 3 achieved a >10-fold reduction but persistently elevated absolute lymphocyte counts (ALCs) of ∼15,000 μl^−1^. Patient 4 had normalization of haematologic parameters with resolution of lymphadenopathy and splenomegaly, but persistent lymphocytosis and marrow disease. Progressive disease (PD) on ibrutinib therapy, characterized by increases in lymphocyte counts with a short lymphocyte-doubling time (<3 months), along with anaemia, thrombocytopaenia and neutropaenia, and recurrence of lymphadenopathy and splenomegaly, was noted after 983, 176 and 554 days, respectively, in Patients 1–3. Patient 4 developed progressive lymphadenopathy, anaemia and thrombocytopaenia without worsening lymphocytosis after 669 days of ibrutinib therapy. Patients 1 and 3 proceeded to other forms of therapy, including anti-CD20 monoclonal antibodies and alternative kinase inhibitors, and were doing well at the time of manuscript preparation (one in remission, one with stable disease), whereas Patient 2 expired from sepsis 63 days after ibrutinib discontinuation, and Patient 4 expired from a haemorrhage 34 days after ibrutinib discontinuation.

### CLL progression interrelates with marked clonal evolution

WES and copy number analysis were performed on two to five serial peripheral blood CLL samples per patient, from which detection of each somatic mutation and inference of its cancer cell fraction (CFF) were undertaken (Supplementary Data 1 and Supplementary Table 1), with the exception of Patient 4, from whom only one sample at time of relapse was available.

**Figure 1:**
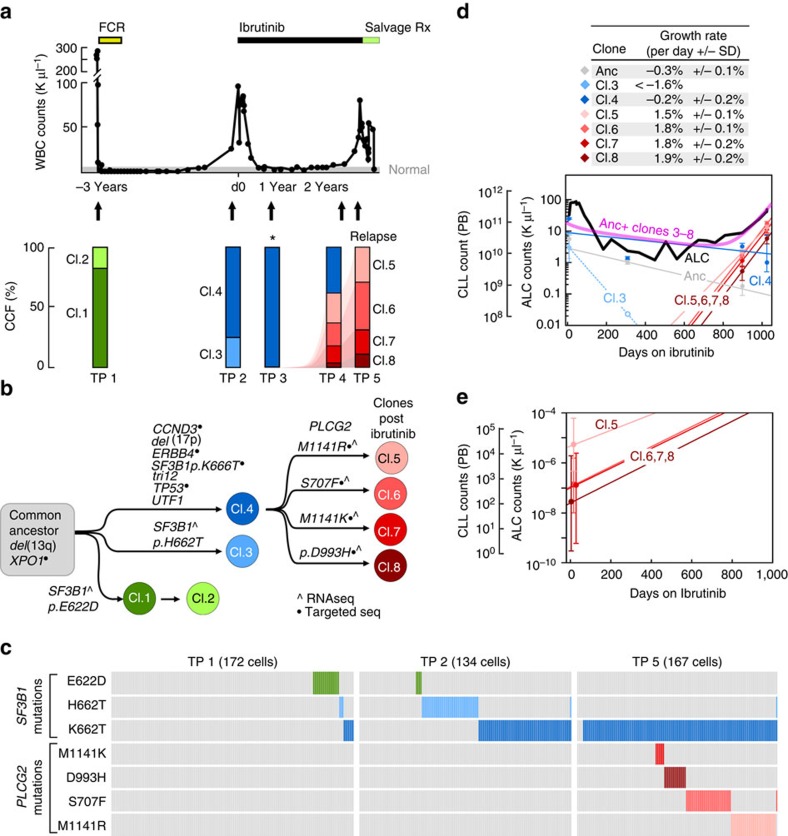
Evidence of clonal evolution with late disease progression following ibrutinib. (**a**) White blood cell counts and treatment course of Patient 1. Peripheral blood specimens were sampled at five time points (indicated by arrows), and CLL cells underwent whole-exome sequencing. Following somatic mutation calling, CCF of somatic variants was inferred by ABSOLUTE analysis of deep-sequencing data of the detected mutations (see Supplementary Figs 1 and 2). Asterisk indicates that this sample had less purity, and hence clone sizes are estimates. (**b**) A phylogenetic tree was inferred based on PHYLOGIC, a novel algorithmic extension of ABSOLUTE. Driver mutations associated with each clone are indicated (a complete listing of somatic mutations and allelic fractions found for each clone in Supplementary Table 2 and Supplementary Figs 1 and 2). (**c**) Multiplexed detection of somatic mutations in 134–172 single cells of Patient 1 at TP1, TP2 (pre-ibrutinib) and TP5 (ibrutinib relapse). Between all three time points, shifting cell subpopulations with SF3B1 mutation are observed. At TP5, SF3B1-K666T is detected in all cells, while the various PLCG2 mutations are detected in distinct subpopulations. Single-cell expression levels of wild-type (WT) and mutated (MUT) PLCG2 are shown in Supplementary Fig. 3B. (**d**) The clonal kinetics during ibrutinib treatment. Filled circles—measurement of the number of cells comprising each subclone at each time point based on the subclone CCF and the corresponding ALCs. Measurements are shown with 95% confidence interval (CI) obtained from posterior distributions of CCFs. Empty circles—upper bound estimates (1% of total CLL cells) for subclones that were below the detection threshold of targeted deep sequencing. Solid lines denote predicted kinetics for clones detected on at least two measurements. Dashed lines represent kinetics with minimal absolute growth rates for clones detected in only one measurement. (**e**) Extrapolation of clone size with 95% CI at the time of treatment initiation for the *PLCG2*-MUT subclones.

Patients 1–3 demonstrated distinct patterns of clonal evolution following exposure to ibrutinib. Patient 1's leukaemia (Fig. 1a,b) was first studied before starting frontline chemoimmunotherapy with FCR 3 years before the start of ibrutinib therapy. The pre-FCR leukaemic population was composed predominantly of a clone harbouring a mutation in *SF3B1* (pG742D), which was eradicated by FCR therapy, and replaced with a clone harbouring biallelic inactivation of *TP53*, trisomy 12 and a new mutation in *SF3B1* (p.K666T; cancer cell fraction (CCF) of 74%). This new clone was present at the time of ibrutinib initiation. Samples during ibrutinib therapy were collected 1, 2 and 2.7 years after initiating therapy. After 2 years of continuous ibrutinib treatment, we observed the emergence of four *PLCG2* mutations (*S707F, M1141R, M1141K* and *D993H*) whose expansion involved the entire sample by 2.7 years and was associated with a rapid rise in ALCs. All of the detected *PLCG2* mutations were novel^6^, although a mutation at the S707 site has been previously implicated in ibrutinib resistance (*S707Y*, ref. 6) and has been shown *in vitro* to disrupt an auto-inhibitory SH2 domain of PLCG2 (ref. 11). We confirmed that these represented four distinct subclones by targeted mutation detection in single cells (Fig. 1c and Supplementary Figs 1 and 2).

We obtained estimates for the absolute numbers of cells in each subclone at each time point by integrating CCF with ALC information (Methods). A model assuming stable growth rates of the clones throughout the period of ibrutinib therapy fits the ALC counts well, and provided estimates of clonal growth rates during treatment. In comparison with the previously estimated growth rate of CLL cells in a heterogeneous group of patients ranging from −0.29 to 0.71% per day^12^, the dominant clone at the start of ibrutinib therapy (clone 4, Fig. 1d) was estimated to decline at a rate of 0.2% (±0.2%) per day, while its progeny clones containing the *PLCG2* mutation grew at a rate of 1.5–1.9%±0.1–0.2% per day. By extrapolating the growth rate back to the time of ibrutinib initiation, we estimated that these four clones were already present at the initiation of therapy (clone size ranging from 140 to 27,000 cells; Fig. 1e).

**Figure 2:**
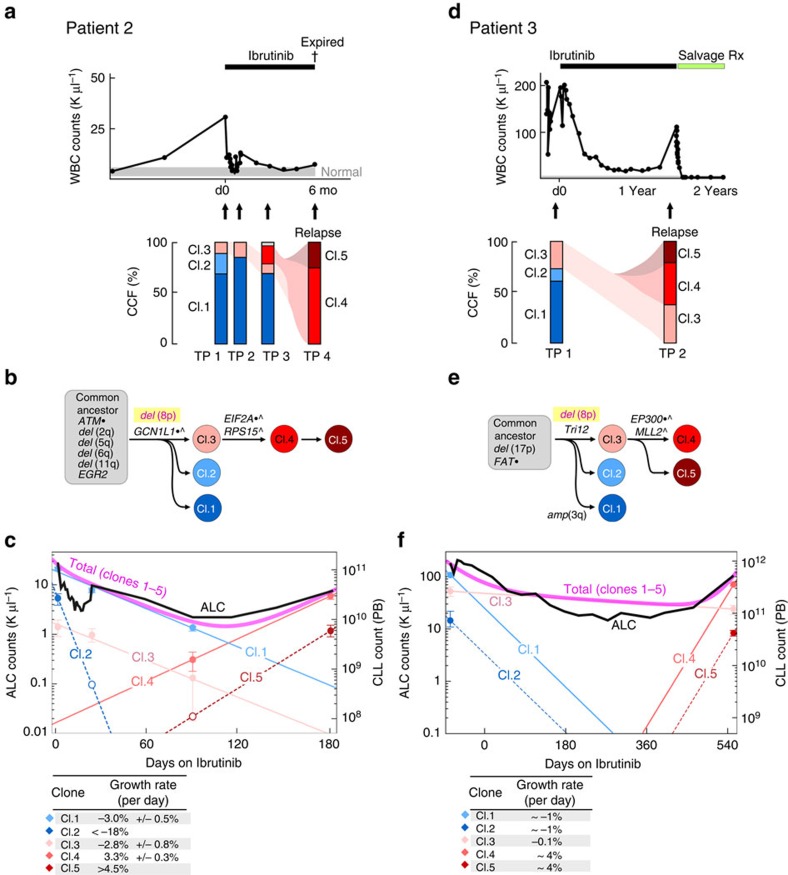
Clonal evolution with early disease progression following ibrutinib. White blood cell counts and treatment courses of Patients 2 (**a**) and 3 (**d**). Peripheral blood specimens were sampled at serial time points (indicated by arrows), and CLL cells underwent whole-exome sequencing. Following somatic mutation calling, CCFs of somatic variants were inferred by ABSOLUTE analysis (Supplementary Figs 1 and 2). The phylogenetic trees for Patients 2 (**b**) and 3 (**e**) were inferred based on Phylogic. Driver mutations associated with each clone are indicated (a complete list of somatic mutations and allelic fractions found for each clone in Supplementary Table 2 and Supplementary Figs 1 and 2). Clonal kinetics during ibrutinib treatment for Patients 2 (**c**) and 3 (**f**). Filled circles—measurements combining clonal fractions and ALC counts. Empty circles are upper bound estimates (1% of total CLL cells) for clones that were below detection. Solid lines denote predicted kinetics for clones with at least two measurements. For Patient 2, dashed lines represent kinetics with minimal absolute growth rates for clones with only one measurement, while for Patient 3 the dashed lines represent kinetics obtained from fitting to ALCs. Measurements are shown with 95% CI obtained from posterior distributions of CCFs. For Patient 3, we assumed clones 1 and 2 have the same rates of decline and clones 4 and 5 have the same growth rates during treatment.

The clinical course of Patients 2 and 3 was notable for a shorter interval until disease progression, which suggests different evolutionary dynamics and resistance profiles^13^. Indeed, in these patients no mutations in *BTK* or *PLCG2* were observed either by WES or by deep sequencing of the known hotspots (*BTK* C481 and *PLCG2* R665), despite average sequencing depths of 1,172 × (range 398–2,263) and 1,126 × (range 354–2,105), respectively. Instead, in the pre-treatment sample of both these patients, a minor subclone harbouring a *del*(8p) was detected, and in both instances the dominant clone at relapse was a progeny of the *del*(8p)-positive minor subclone, after the acquisition of additional putative driver mutations in *EIF2A* (ref. 14) and *RPS15* (refs 15, 16, 17; Patient 2; Fig. 2a,b), and mutations in known hotspots^15^ for the histone acetyltransferase *EP300* (Y1397F) and the chromatin regulator *MLL2* (Q3892; Patient 3; Fig. 2d,e). Growth kinetic analysis of Patient 2 showed the *del*(8p)-containing subpopulation (clone 3) to have a comparable decline rate as the dominant clone at the time of ibrutinib initiation (clone 1; Fig. 2c). However, the progeny of clone 3, which contained the additional mutations in *EIF2A* and *RPS15* (clones 4 and 5), exhibited elevated estimated growth rates of 3.3% and >4.5% per day, respectively, and were estimated to comprise a median of 87,000,000 (or 1 in 1,600) cells at treatment initiation. Patient 3 demonstrated a similar picture (Fig. 2f), with the progeny of the *del*(8p) cells, which contained mutations in *EP300* and *MLL2*, estimated to grow at a rate of ∼4% per day. Together, these findings suggest that the shorter time-to-ibrutinib-failure in Patients 2 and 3 compared with that in Patient 1 was influenced by both the faster growth kinetics and a larger starting population of resistant cells at treatment initiation.

From Patient 4, only one sample at the time of relapse was available, and hence it was not possible to follow changes in the clonal dynamics in this patient. Nonetheless, in this patient, the previously reported *BTK-C481S* mutation was detected by WES, by deep sequencing at the time of relapse and by RNA-sequencing (RNA-seq) of the same sample (Supplementary Fig. 3A).

### Detection of resistant subclones before ibrutinib therapy

To experimentally confirm the calculations of clone size at treatment initiation, we developed an ultrasensitive approach that leverages the ability of droplet-digital amplification technology to evaluate single cells at high throughput. Although bulk quantitative reverse transcriptase PCR (qRT–PCR) of the mutated allele can detect rare mutated transcripts, it cannot provide information on the actual number of affected cells. Deep-targeted sequencing can only affordably detect alleles down to 1 in 100 or 1,000 cells, but is prohibitively expensive for detection of rarer events. Droplet technology, on the other hand, can compartmentalize single cells at very high throughputs (>3,000 per second) inside individual ‘reactors' where enzymatic reactions such as RT–PCR can be performed on each cell.

**Figure 3:**
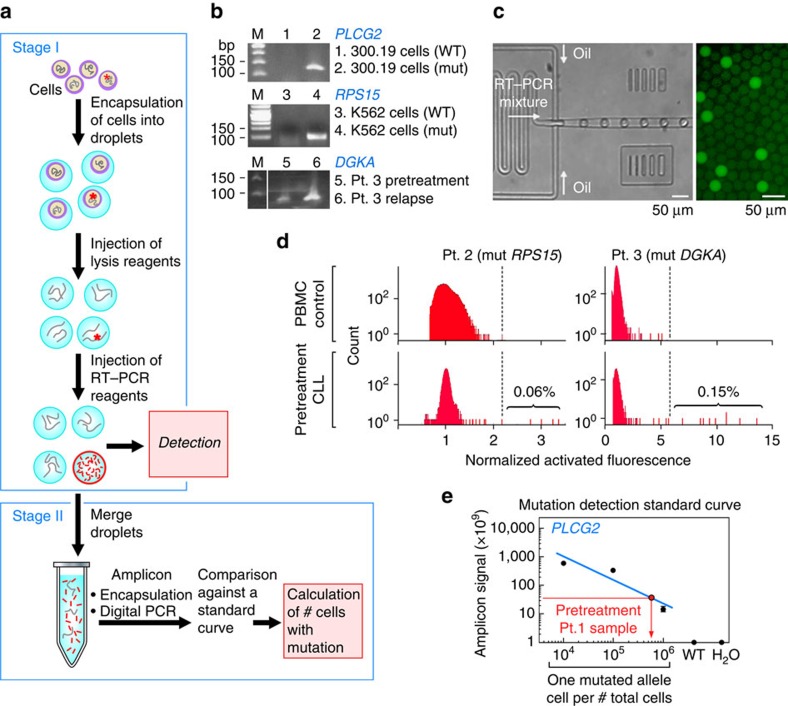
Droplet-based detection of resistance subclones at the time of treatment initiation. (**a**) Schema of the experimental workflow. (**b**) Specificity of the mutation-detection primers visualized on an agarose gel in bulk cell line populations transfected to express minigenes encoding the WT versus mutated (MUT) allele (for *PLCG2* and *RPS15*), or in bulk patient cDNA at pre-treatment and relapse time points (Patient 3, DGKA; **c**). Droplet apparatus, and detection of bright droplets following amplification. (**d**) Detection of MUT *RPS15*-specific single cells in Patient 2 samples and a PBMC control (left) and of MUT *DGKA*-specific single cells in Patient 3 samples and a PBMC control (right). (**e**) Standard curve for the detection of the PLCG2-M1141R template, established based on known input quantities on cell line (murine 30,019 cells, with error bars shown) expressing the MUT template, and detection of *PLCG2-M1141R* in the pre-treatment sample of Patient 1, but not in controls (Supplementary Fig. 4B).

To reliably detect rare mutation-bearing cells, we devised a two-stage amplification and quantification approach (Fig. 3a), focusing on transcripts rather than DNA since the likelihood of single-cell drop-out would be less because of greater transcript abundance. The first stage focuses on the sensitive detection of cDNA from cells harbouring the specified mutated allele. Single cells are encapsulated in droplets, wherein they undergo lysis and the released mutated transcript can efficiently undergo allele-specific RT–PCR (Fig. 3b,c, Supplementary Fig. 3B and Supplementary Table 5). For cell populations of 1 in 10^3^ leukaemia cells or greater, we estimated that this first stage of processing would be sufficient for detection of single mutated cells. Indeed, for Patients 2 and 3, we could detect small cell populations bearing mutations associated with the resistant subclone within the pre-treatment cells, but not in peripheral blood mononuclear cell (PBMC) from normal adult donors (Fig. 3d). Furthermore, these mutation-bearing cells were expanded in number at the time of relapse (Supplementary Fig. 4A). For Patient 2, we detected the presence of mutated *RP515* in 0.06% of pre-treatment cells (calculated previously to be 0.06%), while for Patient 3 we detected mutated *DGKA* in 0.15% cells (not modelled mathematically previously because of too few serial samples). Both measurements were within 1–2 orders of magnitude of detection by deep-targeted sequencing of these mutations (0.57% for Patient 2/mutated *RPS15*; 0.07% for Patient 3/mutated *DGKA*; Supplementary Data 1).

For the detection of rarer populations (such as for the *PLCG2-M1141R* mutation in the pre-treatment sample of Patient 1, calculated to be in 1 in 6 million cells (confidence interval: 1 in 70 million to 1 in 6 million)), we further added the second-stage detection procedure. In this second stage, pooled mutated amplicons were re-encapsulated using a Poisson distribution to ensure that fewer than 30% of droplets contain templates. Following digital PCR of the encapsulated templates, the bright droplets were counted using fluorescence detection (Supplementary Fig. 4B). For *PLCG2-M1141R*, we generated a standard curve from PBMC spiked with known numbers of cells from the 30,019 cell lines, engineered to stably express mutated *PLCG2-M1141R*, and we could reliably detect 1 in 10^4^, 10^5^ and 10^6^ cells with the *PLCG2* mutation compared with 10^6^ cells without the mutation, or the negative water control. In this manner, we confirmed detection of 1 in 500,000 (0.0002%) pre-treatment cells of Patient 1 with mutated *PLCG2-M1141R*—of similar order of magnitude as our mathematical calculations. Altogether, these results confirm that pre-treatment samples already contain resistant subclones before the initiation of targeted inhibition of BTK, albeit at rare frequencies.

### *Trans*-differentiation from CLL to histiocytic sarcoma

**Figure 4:**
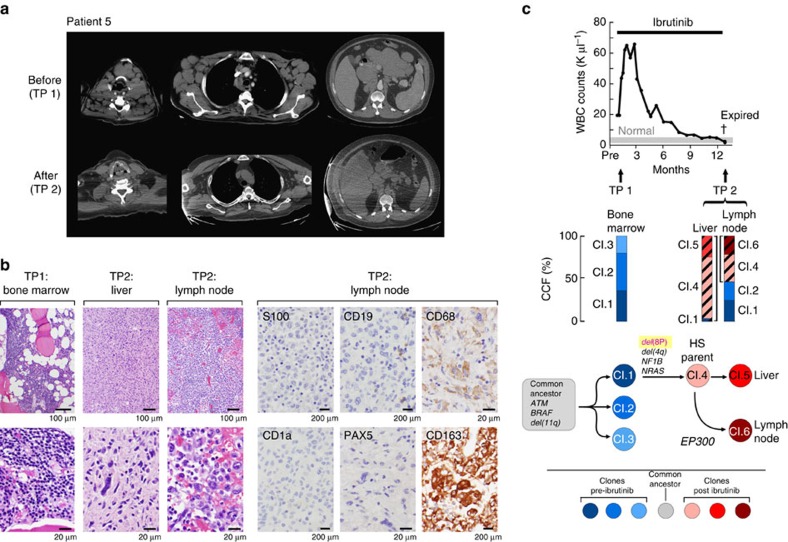
Histiocytic sarcoma *trans*-differentiation of CLL during ibrutinib therapy. (**a**) Regression of lymph node (LN) disease, visualized with CT scan, following ibrutinib exposure (at time point (TP) 2), compared with TP1. (**b**) At TP2 (autopsy), histologic sections of liver and LN, stained with haematoxylin and eosin, showed histiocytic sarcoma with sheets of large atypical cells with irregular shaped nuclei, dense nuclear chromatin and abundant cytoplasm (at × 100 and × 500 inserts). Occasional large neoplastic cells demonstrated one or two prominent eosinophilic nuclei. No lymphoid aggregates were seen. The neoplastic cells within the LN were strongly positive for CD163 and are negative for CD19, CD1a and S100 proteins (all at × 500). (**c**) White blood cell counts and clinical course for Patient 5. Whole-exome sequencing and CCF measurements were made before ibrutinib initiation (TP1) and from post-mortem specimens of the liver and LN (TP2). The fraction of cells that shared the mutations that define the histiocytic sarcoma parent clones are represented with black diagonal lines. Phylogenetic analysis was performed based on PHYLOGIC. A complete list of somatic mutations and allelic fractions for each clone is provided in Supplementary Table 2 and Supplementary Figs 1 and 2.

Patient 5 also demonstrated clonal evolution, but his relapse trajectory was markedly different. At diagnosis, 6 years before the initiation of ibrutinib, this patient presented with bulky lymphadenopathy and *de*l(11q) and *del*(13q) using fluorescence *in situ* hybridization (FISH) analysis. He shortly, thereafter, was treated with frontline FCR, relapsed 2 years later and was re-treated with multiple courses of single-agent fludarabine, rituximab and bendamustine, without any durable responses. Therefore, he proceeded to ibrutinib therapy, and achieved a partial remission, characterized by normalization of the ALC after a transient increase in lymphocytosis and rapid major reduction of his bulky lymph nodes. While still in haematologic remission, he presented on day 392 with a 1-week history of fatigue, malaise, muscle and joint aches, night sweats and low-grade fevers. Evidence for CLL relapse or Richter's transformation was absent since recurrence of bulky lymphadenopathy was not noted on physical examination or CT scans (Fig. 4a), and histopathology examination of bone marrow testing revealed only 2% involvement by CLL cells (compared with 40% before starting ibrutinib therapy). Laboratory studies demonstrated a normal ALC of 1,800 μl^−1^. The patient was admitted for treatment of presumed systemic infection and renal failure and received empiric antibiotics and fluid resuscitation, without improvement. Two days after admission, he was transferred to the ICU for multiorgan dysfunction and expired the same day. Autopsy revealed extensive involvement of the liver, spleen, lung, kidney and multiple lymph nodes with histiocytic sarcoma (HS). The neoplastic cells were positive for monocyte/macrophage markers CD68 (PGM1) and CD163, but negative for CD1a, CD30, CD5, CD15, CD3, CD45 (LCA), CD19, S100 and PAX-5 (Fig. 4b). Immunoglobulin heavy-chain variable region (*IGHV*) gene analysis of HS tissue, which tested negative for any B cells with immunohistochemical, revealed a clonal band (VH3-09), unmutated, which is the same family and somatic mutation status (MS) as originally detected in this patient's CLL cells, indicating clonal relation between the CLL and the HS.

### Genetic dissection of clonal evolution in Patient 5

Three distinct tissues from two time points were evaluated with WES (Fig. 4c). Pre-treatment CLL DNA was extracted from the bone marrow collected before ibrutinib therapy. The progression DNA samples were extracted from lymph node and liver autopsy samples, both of which were confirmed to have involvement by HIS. As germline comparison, DNA was extracted from the uninvolved cardiac muscle. We found that all three samples shared a common set of mutations (for example, *ATM*, *BRAF* and *del*[11q]), indicative of a common ancestor of the CLL and HS, consistent with the *IGHV* analysis. A large CLL subclone (CCF of 36%) distinguished by mutations in *DMBX1* and *DNAJB14* gave rise to the HIS parent clone, which notably contained *del*(8p) as well as an *NRAS* mutation. These mutations define the HS parent as they were shared by HS cells in both the liver and the lymph node samples. Finally, further clonal diversification was observed within the lymph node and the liver samples. For example, all HS cells in the lymph node but not in the liver had the HS parent mutations as well as an *EP300* mutation (N1511S).

### Apoptosis resistance in CLL samples with del(8p)

Having unexpectedly observed *del*(8p) in the resistance clone of three out of five patients with ibrutinib relapse, we examined the genes in this region more closely. The region of *del*(8p) in Patients 2, 3 and 5 encompassed tumour necrosis factor-related apoptosis-inducing ligand-receptor (TRAIL-R) (Supplementary Fig. 5A,B), and we observed a decrease in the TRAIL-R mRNA and protein levels corresponding to an increase in the CCF of the *del*(8p)-harbouring clone (Supplementary Table 3 and Supplementary Fig. 5A). Previous reports have identified haploinsufficiency of the TRAIL-R as a potential target of del(8p)^18^. Intriguingly, a potential indirect mechanism that would link TRAIL resistance to positive selection by the ibrutinib therapy is suggested by the fact that TRAIL concentrations are higher in circulating blood compared with the lymph node environment^19^(Supplementary Fig. 5E). Ibrutinib therapy is known to mobilize CLL cells from the lymph node and spleen to the periphery, resulting in lymphocytosis^6^. Hence, a potential mechanism by which haploinsufficiency of the TRAIL-R could provide a survival advantage for CLL cells with this deletion is through the relative insensitivity of *del*(8p) CLL cells to cell death in the periphery once they are released from the lymph node and are exposed to higher levels of TRAIL. We observed relatively stable high serum levels of TRAIL over time in the CLL patients across the duration of treatment with ibrutinib (Supplementary Fig. 5F).

**Figure 5:**
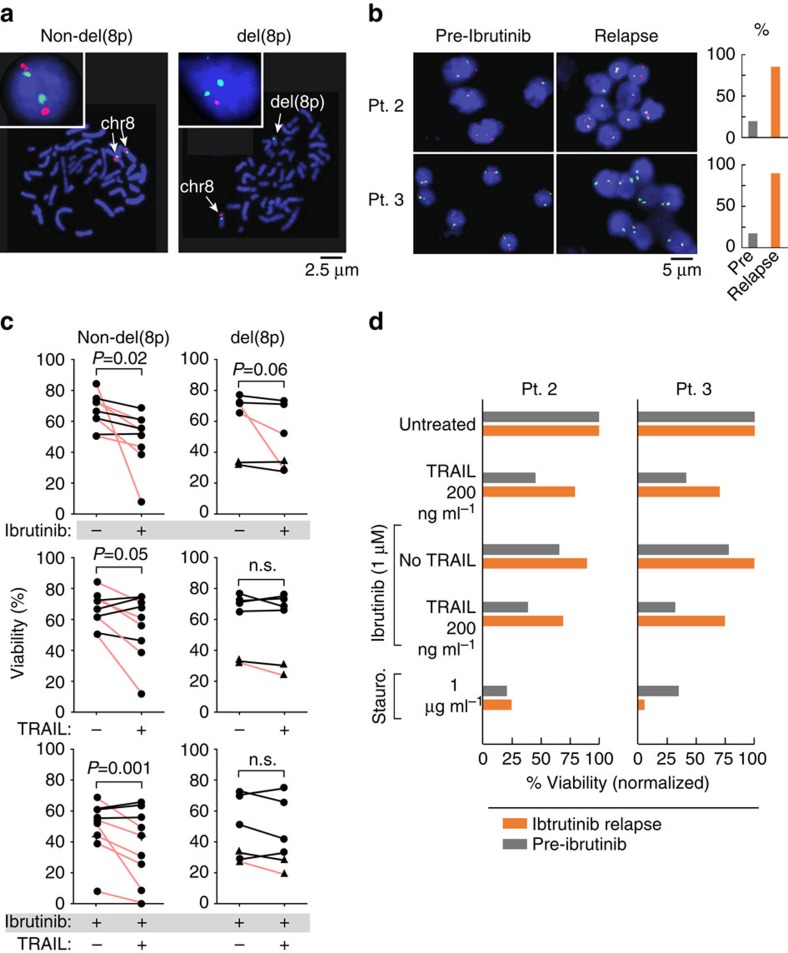
Impact of del(8p) on apoptosis in response to ibrutinib and/or TRAIL in CLL. (**a**) Representative interphase and metaphase FISH results following hybridization for probes specific for chromosome 8p21.3 (red) and chromosome 8 centromere (green), showing a CLL cell with a normal disomic hybridization pattern or with deletion of chromosome 8p (details of the FISH probe in Supplementary Fig. 5a,b,d). (**b**) FISH hybridization of pre-treatment and relapse samples from Patients 2 and 3 to detect del(8p). For each case, 100 nuclei were scored as summarized in the associated bar graphs. (**c**) Primary CLL cells were isolated from peripheral blood and treated with ibrutinib (1 μM) and/or TRAIL (200 ng ml^−1^). Cell death was assessed with Annexin V and propidium iodide (PI) staining and flow cytometry. *P*-values calculated for absolute change in viability. In agreement with the known pleiotropic effects of TRAIL on CLL cells^20^, we found that TRAIL treatment induced apoptosis in six out of nine of non-del(8p) samples, yet could also enhance survival in three out of nine samples. Red—samples with a decrease in cell viability of at least 15% following exposure to TRAIL or ibrutinib. Circle—independent CLL samples, not previously exposed to ibrutinib; triangles—relapse samples from Patients 2 and 3. n.s., not significant. (**d**) Cell viability measurements based on flow cytometric analysis following Annexin V and PI of CLL cells from ibrutinib-resistant Patients 2 and 3 (also see Supplementary Fig. 6a). CLL cells collected before ibrutinib (grey bars) or at the time of ibrutinib resistance (orange bars) were incubated with ibrutinib (1 μM) and/or TRAIL (200 ng ml^−1^) or staurosporine and assessed for viability. At time of ibrutinib resistance, CLL cells were less responsive to TRAIL-induced apoptosis and largely are ibrutinib-resistant, but remained sensitive to staurosporine. Cell viability measurements were equivalent whether based on annexin V/PI or by live cell counts (Supplementary Fig. 6b).

To explore this possibility, we quantified TRAIL- and ibrutinib-induced apoptosis in a mostly independent cohort of six CLL samples with *del*(8p) and in nine non-*del*(8p) controls (*del*(8p) status verified with FISH (Fig. 5a–c and Supplementary Fig. 5D) from patients not previously treated with ibrutinib. Treatment with ibrutinib (1 μM) resulted in a significant decrease in CLL cell viability in non-*del*(8p) controls (*P*=0.02; one-sided paired *t*-test) and to a lesser extent in *del*(8p) samples (*P*=0.06i). In contrast, treatment with TRAIL induced a significant difference in CLL cell viability only in the non-*del*(8p) samples (Fig. 5c). Consistent with previous reports^20^, TRAIL treatment could result in apoptosis or enhanced survival, yet a decrease in viability was observed in a greater proportion of non-*del*(8p) samples (56% versus 17%). In fact, TRAIL treatment induced a 16% median decrease in viability in non-*del*(8p) controls (*P*=0.05; one-sided paired *t*-test) compared with only a 5% median decrease in *del*(8p) CLL samples (*P*=0.12). Hence, monoallelic deletion of chromosome 8p was sufficient to abrogate the positive or negative effects of TRAIL on cell viability *in vitro.* Combination treatment with TRAIL in the presence of ibrutinib further significantly reduced CLL cell viability in non-*del*(8p) samples (*P*=0.01; one-sided paired *t*-test). In contrast, there was only a small, nonsignificant reduction in the median viability by 12% in *del*(8p) CLL samples (*P*=0.13; one-sided paired *t*-test; see Fig. 5c, lower panel). These data indicate that *del*(8p) confers partial resistance to apoptosis in response to TRAIL or TRAIL in combination with ibrutinib. Finally, we sought to determine whether these differences in responses to TRAIL would be evident in serial specimens from two patients developing resistance to ibrutinib via expansion of the *del*(8p) clone. We therefore treated specimens from Patients 2 and 3 with TRAIL and/or ibrutinib (or staurosporine as control) and found the expected sensitivity to TRAIL in the pre-treatment samples and resistance in the relapse samples, confirming the role of *del*(8p) in protection from TRAIL-induced apoptosis (Fig. 5d and Supplementary Fig. 6). Interestingly, ibrutinib-resistant CLL cells remained staurosporine-sensitive, arguing against more general apoptosis resistance and suggesting that kinase inhibitor therapy can further be developed for targeting ibrutinib resistance.

## Discussion

The foremost obstacle to effective targeted therapy is the emergence of disease resistance through clonal evolution. BCR-ABL kinase domain mutations in patients with CML, which confer resistance to the tyrosine kinase inhibitor imatinib^21^, are well-characterized examples for this mechanism. Reminiscent of the imatinib experience, two reports recently highlighted point mutations in *BTK* (C481S)^6^,^7^ that disrupt ibrutinib binding and in its related pathway member *PLCG2* (R665W, L845F, S707Y)^6^ that can activate the BCR pathway independently from BTK as mechanisms of ibrutinib resistance.

An important question emerging from these data is whether ongoing mutagenesis during ibrutinib therapy led to acquisition of these resistance mutations, or whether this is rather because of the expansion of pre-existing subclones under therapeutic pressure. Previous studies failed to detect pre-treatment resistance mutations^6^; however, these were likely limited by the ability of current deep-sequencing approaches to detect minority resistant populations. On the basis of an integrated investigation of the clonal dynamics, growth rate kinetics and experimental detection of rare mutation-bearing cell populations, our analyses support the presence of substantial diversity of resistant subclones at treatment initiation, in line with theoretical predictions^22^. In particular, we observed that the genetic composition and kinetics of ibrutinib resistance were dictated by clone size and growth rate of the resistant cells, that is, the fitness of the resistant clone in relation to other sibling clones^23^.

Patient 1 was particularly exemplary: in this patient, we identified four distinct *PLCG2* mutations, confirmed by RNA-seq and deep-sequencing validation. Shifts in their relative proportion suggest the presence of four distinct subclones, with distinct growth rates. The relatively small proportion of these clones at treatment initiation suggests either no fitness advantage or a minor fitness advantage of these mutations in the absence of ibrutinib, which became accentuated by ibrutinib therapy. This patient's leukaemia had another instance of convergent evolution with clonal shifts in relation to prior FCR therapy, where a clone containing a *SF3B1* mutation was replaced by another clone harbouring a different *SF3B1* mutation (Fig. 1). This case demonstrates the enormous amount of trial and error that occurs in the process of cancer diversification serving its ability to adapt to therapy.

Notably, while the *BTK*-C481S and *PLCG2* mutations were found in two of five subjects, the remaining patients revealed a diverse spectrum of mutations present in resistant cells, illustrating the diversity of mutations participating in the disease progression with ibrutinib therapy. The relapse clones of Patients 2, 3 and 5 arose from parent clones with large deletions of chromosome 8p, previously described to be present in only 5% of the CLL cases and associated with poor prognosis in CLL^24^, especially in patients with del (17p)^25^. *Del*(8p) is also a recurrent event in mantle cell lymphoma and other non-Hodgkin lymphomas. As we previously reported, *del*(8p) likely is a CLL driver that appears later in the evolutionary history of CLL^8^,^24^. This large region encompasses deletions of the TRAIL receptor gene loci^18^, which we confirmed to be downregulated with RNA-seq expression data and with direct flow cytometric detection of surface protein expression from Patients 2 and 3 (Supplementary Table 3 and Supplementary Fig. 5c). Monoallelic deletion of TRAIL-R1/2 (TNFRSF10A/B, also called death receptor 4/5 (DR4/DR5)) can antagonize TRAIL-induced apoptosis in B-NHL^18^, suggesting that TRAIL-R1/2 may function as tumour suppressors. Our functional data support TRAIL receptor haploinsufficiency as a potential resistance mechanism. We noted robust TRAIL-induced apoptosis in CLL samples with intact 8p, but reduced or absent TRAIL-induced apoptosis in samples with *del*(8p), demonstrating that monoallelic deletion was sufficient to abrogate the pro-apoptotic effects of TRAIL. This was confirmed in serial CLL cell samples from Patients 2 and 3, where preserved sensitivity to TRAIL was noted in a pre-treatment sample, and resistance to TRAIL in the relapse sample (Fig. 5d). We also noted additive induction of CLL cell apoptosis by TRAIL in the presence of ibrutinib; this combination may merit further exploration in the clinic. Interestingly, the analysis of clonal kinetics revealed that the *del*(8p) clones initially also declined with ibrutinib therapy (albeit in a slower rate), and subsequently were replaced by their more proliferative progeny, containing additional somatic alterations. This raises the intriguing hypothesis that, while *del*(8p) provides a fitness advantage in the absence of ibrutinib, it needs to cooperate with additional lesions to achieve the resistant phenotype. For example, we identified mutations in *EP300*, *RPS15* or *MLL2* in the cells with del(8p). We recently identified mutations in *RPS15* (all around a hotspot region) as novel significantly recurrent mutations in CLL in a series of 538 CLLs profiled by WES^16^. In addition, Ljungstrom *et al*.^17^ further identified *RPS15* as a recurrently mutated gene in the setting of resistance/relapse to fludarabine-based chemotherapy. Of note, these authors demonstrated that expression of mutant *RPS15* revealed defective regulation of endogenous p53 (with reduced stabilization and increased p53 degradation) compared with wild-type *RPS15*. Both *MLL2* and *EP300* have been identified as significantly mutated across cancers with known roles in histone and chromatin regulation, respectively. How exactly the particular functions of these driver alterations enable bypass of BTK inhibition is unknown. We speculate that alteration of the DNA-damage response and chromatin/histone biology are both paths by which cellular fitness of CLL cells are enhanced, and can lead to BCR-signalling-independent growth.

HISs are myeloid tumours, which rarely evolve in patients with B-NHL and CLL as a result of cross-lineage *trans*-differentiation^26^. As in the case of Patient 5, these exceedingly rare cases of HISs are clonally related to the B-cell malignancy based on shared *IGHV* immunoglobulin gene rearrangements and additional shared mutations. These cases have been interpreted as signs of lineage plasticity of the underlying B-cell neoplasm, a phenomenon that was originally recognized in mouse models^27^. In these models, enforced expression of the transcription factors C/EBPα and C/EBPβ promoted *trans*-differentiation of B cells into macrophages. In humans, *trans*-differentiation of lymphoid malignancies was found to be association with mutations of NRAS and BRAF. Chen *et al*. described a case of Langerhans cell sarcoma, transdifferentiated from CLL that carried a *BRAF* V600E mutation^28^. Buser *et al*. reported about *trans*-differentiation of a T lymphoblastic lymphoma into an indeterminate dendritic cell tumour carrying a G13D mutation of the *NRAS* gene^29^. Interestingly, our patient had both *BRAF* and *NRAS* mutations that may be involved in the *trans*-differentiation process.

The fact that the CLL clone at the time of trans-differentiation remained in remission is characteristic, as is the poor prognosis, with survival generally of only days to weeks. This case of HIS trans-differentiation, with tumour cells no longer dependent on BCR signalling, indicates that ibruitinib resistance may be a more complex process than initially thought, and this potent therapy may serve as a strong evolutionary drive to differentiate away from the B-cell identity and its accompanying dependency on BCR signalling.

We propose a novel analytic framework that is widely applicable across cancer—frequent serial clonal analysis can inform us regarding the clone-specific decline/growth kinetics as they occur in patients. This type of analysis provides vital information regarding the fitness of different genetic lesions with and without therapy, which may be immensely beneficial to the design of the next generation of therapeutic approaches to overcome the evolutionary capacity of cancer.

## Methods

### CLL samples from patients treated with ibrutinib

All five patients were treated at MD Anderson Cancer Center (MDACC) and were enrolled on clinical trials approved by and conducted in accordance with the Institutional Review Board (IRB) of the University of Texas MDACC guidelines and with the principles of the Declaration of Helsinki. Patients 1, 3, 4 and 5 were treated on a phase Ib/II multicentre study of ibrutinib (NCT01105247), while Patient 2 was enrolled on a single-centre phase II clinical trial of ibrutinib and rituximab (NCT01520519). For NCT01105247, the reported rate of resistance at 26 months was 13% (*n*=11)^3^. On NCT01520519, the rate of resistance was 7.5% (*n*=3) at a median follow-up of 16.8 months^10^.

Heparinized blood and/or tissue samples were collected after obtaining informed consent according to the IRB guidelines before and after initiation of ibrutinib therapy, and PMBCs from patient samples were isolated with Ficoll/Hypaque density-gradient centrifugation, cryopreserved with 10% DMSO and stored in vapour-phase liquid nitrogen until the time of analysis. For Patients 1–4, non-lymphoid cells (neutrophils) were isolated by subjecting the non-PBMC cells (following Ficoll separation) to hypotonic erythrocyte lysis^30^. For Patient 5, DNA was extracted from 30-μ sections collected from patient marrow biopsy specimens, obtained as part of routine clinical care, or from formalin-fixed, paraffin-embedded (FFPE) liver, heart and lymph node at the time of autopsy.

### FISH and *IGHV* analyses

The pre-treatment evaluation of all patients included FISH for common CLL chromosome abnormalities by the MDACC clinical laboratory, using a Vysis multicolour probe panel (Abbott Laboratories, Abbott Park, IL) designed to provide simultaneous detection of the 11q22.3 (*ATM* gene) region of chromosome 11; the 17p13.1 (*TP53* gene) region of chromosome 17; the alpha satellite, centromeric region of chromosome 12 (D12Z3); the D13S319 locus (located between RB1 and D13S25 loci) in the 13q14.3 region of chromosome 13; and the 13q34 region (*LAMP1* gene) near the subtelomere of chromosome 13q in two hybridizations (two and three probes per hybridization, respectively), as previously described^31^. A total of 200 interphase cells were analysed for each probe. Positive patient cases were those with 5% or more of cells with the abnormality. Patients' FISH results were categorized according to the Döhner hierarchy^32^. Analysis of the MS of the *IgV*_*H*_ MS was performed in the MDACC clinical laboratory, according to the established protocols described before^33^.

To detect deletions of chromosome 8p, interphase FISH was performed on fixed cell pellets stored at −20 **°**C, obtained from conventional cytogenetic analysis, or cytospins. The cytospins were generated with 5 × 10^4^ CLL cells (Shandon cytospins; 700 r.p.m. for 5 min) fixed with methanol:acetone (3:1) at room temperature for 10 min and then washed with 70% ethanol. Hybridization, using a probe cocktail consisting of Vysis LSI LPL probes targeting 8p21.3 (Abbott Molecular, Des Plaines, IL) and Vysis CEP8 (D8Z2; Abbott Molecular), was performed according to the manufacturer's specifications. One hundred nuclei were scored per slide. Cutoffs for detection of 8p deletion or monosomy 8 were calculated using negative control specimens with matching karyotype information, based on 3 s.d.'s from the mean. The specific cutoff for 8p21.3 deletion was 9.4%.

### Immunohistochemistry

Immunohistochemical stains were performed on FFPE sections of tissue, or bone marrow core biopsies or clots of Patient 5 using the avidin–biotin–peroxidase complex method and an automated immunostainer (Ventana-Biotech, Tucson, AZ). The following antibodies were used:

CD1a(Leica Biosystems, Buffalo Grove, IL, cloneMTB1, prediluted), CD3 (Dako, Carpinteria, CA, clone N/A, dilution1:100), CD5(Labvision Thermo Fisher, Fremont, CA, clone 4C7, dilution 1:20), CD15(BD Biosciences San Jose, CA, clone MMA, dilution 1:40), CD19 (Dako, cloneLE-CD19, dilution 1:100), CD30 (Covance, Dedham, MA, cloneKi-1, dilution 1:80), CD45/LCA (Dako, clone ZB11+PD 7/26, dilution 1:300), CD68/PGM1 (Dako, clone PGM1, dilution 1:450), CD163(Leica Biosystemsclone 10D6, dilution1:100), PAX-5(BD Biosciences, clone 24, dilution 1:35) and S100 protein(BioGenex, Fremont, CA, clone 15E2E2, dilution 1:900). All tissue sections underwent heat-induced antigen retrieval before staining with antibodies. Sequence analysis of the *IGHV* gene region in samples with histologic evidence of HIS (Patient 5) was performed on DNA extracted from FFPE tissue sections. To determine the degree of somatic mutation in the *IGHV* region of non-haematopoietic tissues, patient's *IGHV* sequences were aligned to germline sequences and the patient's known previously characterized *IGHV* sequence (VH3-09), using the international ImMunoGeneTics information system and database tools (IMGT/V-Quest, http://imgt.org). As per convention, the *IGHV* somatic MS was designated as *unmutated* if there was ⩾98% homology, or as *mutated* if there was <98% homology to germline sequences^34^.

### Nucleic acid extraction and quality control

For Patients 1–4, genomic DNA was extracted from CLL PBMC and matched neutrophils (Qiagen, Valencia, CA). DNA analyses were carried out after informed consent under IRB-approved research protocols between MDACC and the Broad Institute. Tumour and normal DNA concentrations were measured using PicoGreen dsDNA Quantitation Reagent (Invitrogen, Carlsbad, CA). For Patient 5 samples, paraffin was removed from samples using Citrisolv and several ethanol washes, and then cells were lysed overnight at 56 °C DNA. After removal of DNA crosslinks through incubation at 90 °C, DNA extraction was performed (QIAamp DNA FFPE Tissue Kit, Qiagen). A minimum DNA concentration of 60 ng ml^−1^ was required for sequencing^35^,^36^. All Illumina sequencing libraries were created with the native DNA. The identities of all tumour and normal DNA samples were confirmed using mass spectrometric fingerprint genotyping of 24 common single-nucleotide polymorphisms (SNPs; Sequenom, San Diego, CA). RNA from CLL—B cells was extracted using standard protocols (RNAeasy kit, Qiagen).

### Whole-exome sequencing

Library construction from CLL and matched germline DNA of Patients 1–5 was performed as described in ref. 37, with the following modifications: (i) initial genomic DNA input into shearing was reduced from 3 μg to 10–100 ng in 50 μl of solution; (ii) for adapter ligation, Illumina paired-end adapters were replaced with palindromic forked adapters (from Integrated DNA Technologies), with unique eight-base molecular barcode sequences included in the adapter sequence to facilitate downstream pooling. With the exception of the palindromic forked adapters, the reagents used for end repair, A-base addition, adapter ligation and library enrichment PCR were purchased from KAPA Biosciences in 96-reaction kits, (iii) during the post-enrichment solid-phase reversible immobilization (SPRI) cleanup, elution volumes were reduced to 20 μl to maximize library concentration, and a vortexing step was added to maximize the amount of template eluted. Any libraries with concentrations below 40 ng μl^−1^ (per PicoGreen assay, automated on an Agilent Bravo) were considered failures and reworked from the start of the protocol. Following library construction, hybridization and capture were performed using the relevant components of Illumina's Rapid Capture Exome Kit and following the manufacturer's suggested protocol. All hybridization and capture steps were automated on the Agilent Bravo liquid-handling system. On the basis of qPCR quantification with probes specific to the ends of the adapters (KAPA Biosystems), libraries were normalized to 2 nM, and then denatured using 0.1 N NaOH on the Perkin-Elmer MiniJanus. After denaturation, libraries were diluted to 20 pM (hybridization buffer, Illumina).

Cluster amplification of denatured templates was performed according to the manufacturer's protocol (Illumina) using HiSeq v3 cluster chemistry and HiSeq 2500 flowcells to an average sequencing depth of 138 × (Supplementary Table 1). Flowcells were sequenced on HiSeq 2500 using v3 Sequencing-by-Synthesis chemistry, and then analysed using RTA v.1.12.4.2 or later. Each pool of whole-exome libraries was run on paired 76-bp runs, and an eight-base index-sequencing read was performed to read molecular indices across the number of lanes needed to meet coverage for all libraries in the pool. Alignments to hg19 using bwa version 0.5.9-r16 (ref. 38) and quality control were performed using the Picard (http://picard.sourceforge.net/) and Firehose (http://dx.doi.org/10.7908/C180514N) pipelines at the Broad Institute. Firehose is a framework combining workflows for the analysis of cancer-sequencing data. The workflows perform quality control, local re-alignment, mutation calling, small insertion and deletion identification, rearrangement detection and coverage calculations, among other analyses. Average coverage is listed in Supplementary Table 1. WES data have been deposited in dbGaP (phs001091.v1.01).

### Mutation calling

The MuTect algorithm (http://www.broadinstitute.org/cancer/cga/mutect) was used to identify somatic mutations in targeted exon data^31^. MuTect identifies candidate somatic mutations by Bayesian statistical analysis of bases and their qualities in the tumour and normal BAM files at a given genomic locus. The lowest allelic fraction at which somatic mutations could be detected on a per-sample basis was estimated based on cross-contamination level of 2%. All somatic mutations were reviewed manually using the Integrative Genomics Viewer^32^.

### Somatic copy number alteration identification

Somatic copy number alterations (SCNAs) were identified from the analysis of genome-wide copy number profiles of CLL and matched germline DNA, obtained using the Genomewide Human SNP Array 6.0 (Affymetrix) according to the manufacturer's protocol (Genetic Analysis Platform, Broad Institute, Cambridge, MA). SNP array data were deposited in dbGaP (phs000435.v1.p1). Alternatively, SCNAs were inferred from the whole-exome data from the ratio of tumour read depth to the expected read depth derived from a panel of normal samples using the ReCapSeg programme, which is available for download (http://gatkforums.broadinstitute.org/gatk/categories/recapseg-documentation). Allele-specific analysis allowed for the identification of copy neutral loss of heterozygosity (LOH) events and quantification of the homologous copy ratios using both Hapseg^33^ on SNP arrays and Allelic CapSeg on exomes. Regions with germline copy number variants were excluded from the analysis.

### Deep sequencing of somatic single-nucleotide variants

When DNA was available (Supplementary Table 1), deep sequencing was performed by targeted resequencing using microfluidic PCR (Access Array System, Fluidigm). In total, 112/133 candidate somatic mutations identified in Patients 1–4 were sequenced with this approach (Supplementary Table 3). Tumour and matched normal samples were included in this analysis to exclude germline variants. Target-specific primers were designed to flank sites of interest and produce amplicons of 200±20 bp. Per well, molecularly barcoded, Illumina-compatible specific oligonucleotides containing sequences complementary to the primer tails were added to the Fluidigm Access Array chip together with genomic DNA samples (20–50 ng of input) such that all amplicons for a given DNA sample shared the same index, and PCR was performed according to the manufacturer's instructions. From each individual collection well from the Fluidigm chip, indexed libraries were recovered for each sample, quantified using picogreen and then normalized for uniformity across libraries. Resulting normalized libraries were loaded on the MiSeq instrument and sequenced using paired-end 150-bp sequencing reads^39^. The mean coverage per sample is listed in Supplementary Table 1 (range 938.9–5419.5 × ).

### ABSOLUTE analysis and clonal evolution mapping

ABSOLUTE pipeline was implemented as previously described^8^,^34^ to the sequencing data to convert allelic fractions to CFFs, accounting for sample purity and the local copy number information. The CCFs were clustered as previously described^8^ to delineate distinct subclonal populations. Phylogenetic relationships between these populations were inferred using patterns of shared mutations and CCF, as previously described^40^. The CCF of each clone was converted to clonal size (in cell number) by multiplying the CCF by the total size of the circulating CLL population (as measured by the ALCs per microlitre times the total blood volume). Clone-specific growth/decline rates were then inferred by using regression applied to the measurements available for each subclone, assuming fixed exponential growth rates.

### Mathematical analysis of clonal kinetics

CCFs obtained from the ABSOLUTE analysis were combined with ALC counts to obtain estimates for the numbers of cancer cells in each clone present at the time of sequencing, assuming 5 l as the peripheral blood volume^41^. We assumed that during treatment clones either grow or decline exponentially, with constant rates. For clones with exactly two measurements, s.d.'s of growth rates were estimated using posterior distributions of CCFs. For clones with more than two measurements, we report s.e.'s for growth rates obtained from linear regression in the log domain. We estimated the number of cells in a resistant clone at the time of initiation of ibrutinib treatment under the assumption that the growth rate of the resistant clone remains constant during treatment. Confidence intervals are obtained using posterior distributions of CCFs.

### Droplet detection of single cells with somatic gene mutations

CLL cells, PBMC or cell lines resuspended in RPMI 1640 with 20% fetal bovine serum were applied to polydimethylsiloxane microfluidic devices that were fabricated using standard soft lithographic methods^42^. These microfluidic chips contain a co-flow droplet generator (cross-section of 35 μm^2^) to yield 50-μm monodisperse aqueous drops in fluorinated oil, HFE-7500 (3M, St Paul, MN) containing 2% (w/w) Krytox-PEG diblock co-polymer surfactant (RAN Biotech, Beverly, MA). The microfluidic channel walls were rendered hydrophobic by treating them with Aquapel (PPG, Pittsburgh, PA). Cell lysis buffer (2 × , 1 M Tris-HCl pH 8.0, 10% Tween-20 and 100 mg ml^−1^ proteinase K in one channel and a suspension of a single-cell population or mixtures of cell populations are encapsulated together in drops via co-flow at a 1:1 ratio. The droplets were collected in 200 μl in a PCR tube and covered with mineral oil. Cell lysis within the drops was achieved using the following conditions: 37 °C for 10 min, 50 °C for 20 min and 70 °C for 10 min. Subsequently, the droplets containing single lysed cells were maintained on ice.

To amplify transcripts with the mutated alleles, the droplet suspension (at 33 pl volume per droplet) was introduced into a microfluidic pico-injection device and injected droplet by droplet with 50 μl of a 2 × RT–PCR cocktail through electrocoalescence^43^. The 2 × RT–PCR cocktail contained 4 μl of OneStep RT–PCR enzyme mix with 2 × OneStep RT–PCR buffer (Qiagen) 800 μM dNTPs, 0.6 μM forward and reverse primers for patient-specific somatic mutations (purchased from IDT, Coralville, Iowa), 0.5 μM Taqman probe (Life Tech, Grand Island, NY), 0.4 μg μl^−1^ BSA and 0.4% Tween-20 (see Supplementary Table 5 for primer and probe sequences). Droplets were spaced on the chip by oil with 2% w/w surfactant. The device electrodes were connected to a high-voltage TREK 2210 amplifier (TREK, Lockport, NY), which supplies a 100-V sine wave at a frequency of 25 kHz. The flow rate of the PCR cocktail was chosen to ensure that the buffer would be added at ∼1:1 ratio on coalescence. Typical flow rates fulfilling these requirements were 300 μl h^−1^ for oil with surfactant, 60 μl h^−1^ for the droplets containing lysed cells and 30 μl h^−1^ for the PCR cocktail. The droplets were collected in a PCR tube and covered with mineral oil to prevent evaporation. RT–PCR was performed using the following conditions: 50 °C for 30 min, 95 °C for 10 min, two cycles of 94 °C for 15 s and 64 °C for 8 min, and 38 cycles of 95 °C for 15 s and 62 °C for 1 min.

Amplified mutated transcripts within single cells were detected with microfluidic-based sorting and signal detection. We re-injected the post-amplification drops, achieving a stream of evenly spaced drops through co-flowing of the drop suspension (flow rate 15 μl h^−1^) and HFE-7500 oil with 1% surfactant (flow rate 180 μl h^−1^) into a ‘T' junction. This stream flowed through a 25 × 25-μm channel, and was exposed to an excitation laser (488 nm). Fluorescence information from single cells was collected with a microscope objective and focused to a photomultiplier tube (Hammamatsu). The pulses were acquired by a real-time field-programmable gate array card (National Instruments, Austin, TX), recorded by a LabView programme and analysed in MATLAB. The pulse height was used as the measure of droplet fluorescence. The pulse width, which is the duration of time for a drop to pass through the laser, was used as the measure of droplet size. The sensitivity of our photomultiplier tube was sufficiently high to detect droplets not containing target templates because of the intrinsic fluorescence of the Taqman probe. Cells were designated as positive of the normalized activated fluorescence was higher than the signal generated by control PBMC for healthy adult volunteers.

For detection of very rare cells, we developed a second step of droplet analysis using digital PCR. To obtain the templates for the second-round digital PCR, 25 μl of 1H,1H,2H,2H-perfluoro-1-octanol (PFO; Sigma-Aldrich, St Louis, MO) was added to the pool of emulsion droplets and gently centrifuged to separate the phases, such that the PCR products from the first-round RT–PCR were in the liquid phase. PCR products were then diluted 1,000-fold, and 1 μl of the resulting product was encapsulated at a single template per droplet using a microfluidic device that contains a flow-focusing droplet maker with a cross-section of 15 × 25 μm to generate 25-μm monodisperse aqueous drops in HFE-7500 containing 2% (w/w) surfactant. The flow is driven by applying a −0.4 PSI vacuum at the outlet. The templates were then amplified using a 25-μl PCR cocktail containing 1 μl of OneStep RT–PCR enzyme mix with 1 × OneStep RT–PCR buffer (Qiagen), 400 μM dNTPs, 0.25 μM forward and reverse primers, 0.24 μM Taqman probe, 0.2 μg μl^−1^ BSA and 0.2% Tween-20 using the following RT–PCR protocol: 95 °C for 10 min, 40 cycles of two cycles of 94 °C for 15 s, 64 °C for 8 min and 38 cycles of 95 °C for 15 s, 62 °C for 1 min. To quantify the mutant cells in the original sample, we compared fluorescence obtained from the experimental sample against a standard curve generated by the fluorescence detection from known mixtures of specific cell lines generated to express the gene of interest with or without the mutation of interest (see Supplementary Table 4 for conditions of cloning these plasmids; Fig. 3e).

### RNA-seq

For generation of RNA libraries, 5 μg of total RNA was poly-A-selected using oligo-dT beads to extract the desired mRNA, treated with DNase and then processed with SPRI beads as per the manufacturer's protocol. The selected Poly-A RNA was then fragmented into 450-bp fragments in an acetate buffer at high heat. Fragmented RNA was SPRI-cleaned and primed with random hexamers before first-strand cDNA synthesis. To prevent hair-pinning, the first strand was reverse-transcribed off the RNA template in the presence of Actinomycin D, followed by SPRI bead purification. The RNA in the RNA–DNA complex was then digested using RNase H. The second strand was next synthesized with a dNTP mixture, in which dTTPs were replaced with dUTPs. After another round of SPRI bead purification, the resultant cDNA was processed per Illumina library construction according to the manufacturer's protocol (end repair, phosphorylation, adenylation and adaptor ligation with indexed adaptors). SPRI-based size selection was performed to remove adaptor dimers present in the newly constructed cDNA library. Libraries were treated with uracil-specific excision reagent to nick the second strand at every incorporated uracil (dUTP). Subsequently, libraries were enriched with eight cycles of PCR using the entire volume of sample as template. Following enrichment, the library was quantified using picogreen, and the fragment size is measured (Agilent Bioanalyzer). Samples were pooled and sequenced using either 76 or 101 bp paired-end reads. RNA-seq BAMs were aligned to the hg19 genome using the TopHat suite. Each somatic base substitution detected by WES was compared with reads at the same location in RNA-seq. On the basis of the number of alternate and reference reads, a power calculation was obtained with beta-binomial distribution (power threshold used was greater than 80%). Mutation calls were deemed validated if two or greater alternate allele reads were observed in RNA-seq at the site, as long as RNA-sseq was powered to detect an event at the specified location (power >0.8). RNA-seq data were deposited in dbGaP (phs001091.v1.01).

### Generation of stable cell lines

To generate stable cell lines expressing wild-type and mutant *PLCG2* and *RPS15*, cDNA fragments around the mutation sites of interest were cloned (see Supplementary Table 4 for mutation sites, primers and vectors). Mutations were introduced into the cDNA fragments through site-directed mutagenesis (Quickchange II Site-Directed Mutagenesis Kit, 200523-5, Agilent Technology). The vectors were linearized by MfeI, and transfected into murine 300.19 cells through electroporation. The transfected cells were selected with antibiotics for 2 weeks to generate the stable cell lines.

### Microfluidics-based single-cell detection of mutations

Fluorescence-activated cell sorting-sorted CD19^+^CD5^+^7AAD^−^ single cells were collected and processed through the pre-amplification step as described in ref. 44, with the exception that Reverse Transcription Master Mix (Fluidigm 100-6297) was used in the reverse transcriptase step and 5 × PreAmp Master Mix (Fluidigm 100-5744) was used in the pre-amplification step. The use of a 5 × formulation enabled reducing the volume of the pre-amplification reaction to 10 μl, which enhanced sensitivity. Paired mutated- and normal-allele-specific primers were designed using a nested design, with outer primers for pre-amplification and inner primers for qPCR detection, such that amplification of the mutated alleles with the two assays yielded a difference of at least six cycles. Each assay consisted of an allele-specific SuperSelective primer^45^ and a common primer shared by the normal and mutation assay. The sequences of the primers used are provided in Supplementary Table 5. Single-cell cDNA was submitted for multiplexed pre-amplification with a mixture of all the outer primers for patient-specific, mutation-specific assays at a final concentration of 50 nM each primer. Pre-amplified cDNA samples from single cells were then analysed with qPCR using 96.96 Dynamic Array integrated fluidic circuits (IFCs) and the Biomark HD System from Fluidigm as per the manufacturer's procedures. To detect somatic mutations, a Master Mix was prepared consisting of Fast-Plus EvaGreen Master Mix (2 × , 420 μl) with Low ROX (Biotium 31014), DNA-binding dye sample loading reagent (20 × , 42 μl), 500 mM EDTA (1.5 μl) and H_2_O (16.5 μl) with 4 μl of this mix dispensed per well of a 96-well assay plate. Three microlitres of pre-amplified cDNA sample were added to each well, and the plate was briefly vortexed and centrifuged. Following priming of the IFC in the IFC Controller HX, 5 μl of the cDNA sample+Master Mix were dispensed to each Sample Inlet of the 96.96 IFC. After loading the assays and samples into the IFC Controller HX, the chip was transferred to the Biomark HD and PCR was performed (using protocol GE Fast 96 × 96 PCR+Melt v10.pcl). The thermal cycling protocol consists of a Thermal Mix of 70 °C, 40 min; 66 °C, 30 s, hot start at 95 °C, 2 min, PCR cycle of two cycles of 96 °C, 5 s; 64 °C, 480 s, PCR cycle of 30 cycles of 96 °C, 5 s; 62 °C, 30 s and melting using a ramp from 60 to 95 °C at 1 °C per 3 s. Data were analysed processing with the Fluidigm Real-Time PCR Analysis software, which uses the Linear (Derivative) Baseline Correction Method and the Auto (Global) Ct Threshold Method. For each patient sample, two independent IFCs were run and the results consolidated by averaging the technical replicates.

To call mutations, we first modelled the background level of expression of the mutated allele by linear regression through assessment of normal B cells known to have absence of the mutation of interest. We then calculated the fraction of the normalized mutant allele over normal plus normalized mutant allele. Cells with this normalized fractional mutant allele below 0.15 were called as ‘normal', while cells with this normalized fractional mutant allele greater than 0.3 were called as ‘mutant', and anything in between were called as ‘unclear.' A threshold of 0.3 was determined by *ad hoc* assessment on the negative controls. We restricted subsequent analysis to cells for which we could confidently call ‘normal' or ‘mutant' status. Cells for which we did not detect either the mutant or the normal alleles, yielding a normalized mutant allele level of 0/0, were excluded.

### *In vitro* viability experiments

After obtaining informed consent, peripheral blood samples were obtained from patients fulfilling diagnostic and immunophenotypic criteria for CLL at MDACC or at Dana-Farber Cancer Institute (DFCI). Consent for samples used in this study was obtained in accordance with the Declaration of Helsinki on protocols that were reviewed and approved by the IRBs of MDACC or Dana-Farber/Harvard Cancer Center. *In vitro* testing of cell viability of CLL cells with or without del(8p) (verified with a FISH-specific probe) following exposure to ibrutinib and/or TRAIL was performed by flow cytometry using Annexin V and propidium iodide staining. Fresh or thawed cryopreserved mononuclear cells were treated with 1 μM ibrutinib (Selleck Chemicals, Houston, TX) and/or Super Killer TRAIL (ENZO Biochem, New York, NY), and cell viability was assessed in CD19-positive CLL cells at 24-h intervals on an LSR Fortessa flow cytometer (BD Biosciences, San Diego, CA). Data analysis was performed on the time point for each sample that exhibited viability closest to 75% in untreated cells. Cell viability was measured by % viable (Annexin V (BD Biosciences) and propidium iodide (Sigma, St Louis, MO) negative) or absolute live cell counts. Absolute live cell counts were obtained by supplementing cells before flow cytometric analysis with CountBright Absolute Counting Beads (Life Technologies) and acquiring each sample based on bead count. The two different methods of cell viability analysis yielded comparable results (Supplementary Fig. 6B). Data were presented as % viable, unless otherwise indicated. Cell surface TRAIL expression was detected using the allophycocyanin (APC)-conjugated anti-human CD261 (DR4) antibody (clone DJR1, Biolegend, San Diego, CA).

### TRAIL plasma levels

TRAIL plasma levels were measured using Quantikine solid-phase ELISA Kits according to the manufacturer's instructions (R&D Systems, Minneapolis, MN). The absorbance was recorded (ELx808 microplate reader, Bio-Tek Instruments), and data collection and analysis were performed using the Gen5 software Version 1.08 (Bio-Tek Instruments). Plasma samples were collected in tubes containing EDTA as an anticoagulant. As per the manufacturer, the mean normal TRAIL level in plasma is 82 pg ml^−1^ (range: 34–163 pg ml^−1^).

## Additional information

**Accession codes:** The whole-exome sequencing and RNA-seq data have been deposited in dbGaP under accession code phs001091.v1.01.

**How to cite this article:** Burger, J. A. *et al*. Clonal evolution in patients with chronic lymphocytic leukaemia developing resistance to BTK inhibition. *Nat. Commun.* 7:11589 doi: 10.1038/ncomms11589 (2016).

## Supplementary Material

Supplementary Information Supplementary Figures 1-6 and Supplementary Tables 1-4.

Supplementary Data 1 Mutation annotation file including cancer cell fraction estimates

## Figures and Tables

**Table 1:** Patient characteristics.

**Pt number**	**Age (years)/gender/Rai stage**	**Prior therapy**	**Pre-ibrutinib FISH cytogenetics**	**IGHV (M, U)**	**Treatment**	**Best response to ibrutinib**	**Time to PD on ibrutinib**
**1**	59/M Rai III	FCR	del (17p), del (13q)	ND	Ibrutinib	PR	983
**2**	36/F Rai IV	FCR, R+HDMP	del (11q)*	U	Ibrutinib +rituximab	PR	176
**3**	85/F Rai IV	R, BR, CLB, R+HDMP	del (17p), del (13q), trisomy 12*	U	Ibrutinib	PR	554
**4**	58/M Rai IV	FCR, FR, CHOP, allo-Tx, BR, revlimid, ofatumumab	del (17p), del (11q), del (13q)*	U	Ibrutinib	PR	669
**5**	58/M Rai II	FCR, F, R, B	del (11q), del (13q)	U	Ibrutinib	PR	392

allo-Tx, allogeneic stem cell transplantation; BR, bendamustine, rituximab; CHOP, cyclophosphamide, doxorubicin, vincristine and prednisone; CLB, chlorambucil; F, female; (prior therapy) FCR, fludarabine, cyclophosphamide, rituximab; FISH, fluorescence *in situ* hybridization; F, R, B, single-agent fludarabine, rituximab, bendamustine; FR, fludarabine, rituximab; IGHV, immunoglobulin heavy-chain variable region genes; M, male; M, mutated; ND, not determined; PD, progressive disease; (best response) PR, partial remission; Pt, patient; R+HDMP, rituximab+high-dose methylprednisolone; U, unmutated.

^*^Complex cytogenetics pre-ibrutinib: Pt 2: 46,XX,del(2q37),del(6q21q23), add(7q36),del(11q21q25); Pt 3: 45,X,add(X)(q24), add(6p23), add(12p13), del(17p11.2), -21; Pt 4: 45,XY,del(6q13q23), add(8p21), del(11q21q24), -17,-18,+mar[5].

## References

[b1] Burger J. A. & Chiorazzi N. B cell receptor signaling in chronic lymphocytic leukemia. Trends Immunol. 34, 592–601 (2013). 23928062 10.1016/j.it.2013.07.002 PMC3898793

[b2] Friedberg J. W. . Inhibition of Syk with fostamatinib disodium has significant clinical activity in non-Hodgkin lymphoma and chronic lymphocytic leukemia. Blood 115, 2578–2585 (2010). 19965662 10.1182/blood-2009-08-236471 PMC2852362

[b3] Byrd J. C. . Targeting BTK with ibrutinib in relapsed chronic lymphocytic leukemia. N. Engl. J. Med. 369, 32–42 (2013). 23782158 10.1056/NEJMoa1215637 PMC3772525

[b4] Furman R. R. . Idelalisib and rituximab in relapsed chronic lymphocytic leukemia. N. Engl. J. Med. 370, 997–1007 (2014). 24450857 10.1056/NEJMoa1315226 PMC4161365

[b5] Honigberg L. A. . The Bruton tyrosine kinase inhibitor PCI-32765 blocks B-cell activation and is efficacious in models of autoimmune disease and B-cell malignancy. Proc. Natl Acad. Sci. USA 107, 13075–13080 (2010). 20615965 10.1073/pnas.1004594107 PMC2919935

[b6] Woyach J. A. . Resistance mechanisms for the Bruton's tyrosine kinase inhibitor ibrutinib. N. Engl. J. Med. 370, 2286–2294 (2014). 24869598 10.1056/NEJMoa1400029 PMC4144824

[b7] Furman R. R. . Ibrutinib resistance in chronic lymphocytic leukemia. N. Engl. J. Med. 370, 2352–2354 (2014). 24869597 10.1056/NEJMc1402716 PMC4512173

[b8] Landau D. A. . Evolution and impact of subclonal mutations in chronic lymphocytic leukemia. Cell 152, 714–726 (2013). 23415222 10.1016/j.cell.2013.01.019 PMC3575604

[b9] Thompson P. A. . Complex karyotype is a stronger predictor than del(17p) for an inferior outcome in relapsed or refractory chronic lymphocytic leukemia patients treated with ibrutinib-based regimens. Cancer 121, 3612–3621 (2015). 26193999 10.1002/cncr.29566 PMC4866653

[b10] Burger J. A. . Safety and activity of ibrutinib plus rituximab for patients with high-risk chronic lymphocytic leukaemia: a single-arm, phase 2 study. Lancet Oncol. 15, 1090–1099 (2014). 25150798 10.1016/S1470-2045(14)70335-3 PMC4174348

[b11] Zhou Q. . A hypermorphic missense mutation in PLCG2, encoding phospholipase Cgamma2, causes a dominantly inherited autoinflammatory disease with immunodeficiency. Am. J. Hum. Genet. 91, 713–720 (2012). 23000145 10.1016/j.ajhg.2012.08.006 PMC3484656

[b12] Messmer B. T. . In vivo measurements document the dynamic cellular kinetics of chronic lymphocytic leukemia B cells. J. Clin. Invest. 115, 755–764 (2005). 15711642 10.1172/JCI23409 PMC548318

[b13] Chiron D. . Cell-cycle reprogramming for PI3K inhibition overrides a relapse-specific C481S BTK mutation revealed by longitudinal functional genomics in mantle cell lymphoma. Cancer Discov. 4, 1022–1035 (2014). 25082755 10.1158/2159-8290.CD-14-0098 PMC4155003

[b14] Fritsch R. M., Schneider G., Saur D., Scheibel M. & Schmid R. M. Translational repression of MCL-1 couples stress-induced eIF2 alpha phosphorylation to mitochondrial apoptosis initiation. J. Biol. Chem. 282, 22551–22562 (2007). 17553788 10.1074/jbc.M702673200

[b15] Lawrence M. S. . Discovery and saturation analysis of cancer genes across 21 tumour types. Nature 505, 495–501 (2014). 24390350 10.1038/nature12912 PMC4048962

[b16] Landau D. A. . Mutations driving CLL and their evolution in progression and relapse. Nature 526, 525–530 (2015). 26466571 10.1038/nature15395 PMC4815041

[b17] Ljungstrom V. . Whole-exome sequencing in relapsing chronic lymphocytic leukemia: clinical impact of recurrent RPS15 mutations. Blood 127, 1007–1016 (2016). 26675346 10.1182/blood-2015-10-674572 PMC4768426

[b18] Rubio-Moscardo F. . Characterization of 8p21.3 chromosomal deletions in B-cell lymphoma: TRAIL-R1 and TRAIL-R2 as candidate dosage-dependent tumor suppressor genes. Blood 106, 3214–3222 (2005). 16051735 10.1182/blood-2005-05-2013

[b19] Herbeuval J. P. . HAART reduces death ligand but not death receptors in lymphoid tissue of HIV-infected patients and simian immunodeficiency virus-infected macaques. AIDS 23, 35–40 (2009). 19050384 10.1097/QAD.0b013e32831cb907 PMC7337596

[b20] Secchiero P. . Aberrant expression of TRAIL in B chronic lymphocytic leukemia (B-CLL) cells. J. Cell Physiol. 205, 246–252 (2005). 15887227 10.1002/jcp.20392

[b21] Shah N. P. . Multiple BCR-ABL kinase domain mutations confer polyclonal resistance to the tyrosine kinase inhibitor imatinib (STI571) in chronic phase and blast crisis chronic myeloid leukemia. Cancer Cell 2, 117–125 (2002). 12204532 10.1016/s1535-6108(02)00096-x

[b22] Bozic I. & Nowak M. A. Timing and heterogeneity of mutations associated with drug resistance in metastatic cancers. Proc. Natl Acad. Sci. USA 111, 15964–15968 (2014). 25349424 10.1073/pnas.1412075111 PMC4234551

[b23] Komarova N. L., Burger J. A. & Wodarz D. Evolution of ibrutinib resistance in chronic lymphocytic leukemia (CLL). Proc. Natl Acad. Sci. USA 111, 13906–13911 (2014). 25201956 10.1073/pnas.1409362111 PMC4183279

[b24] Brown J. R. . Integrative genomic analysis implicates gain of PIK3CA at 3q26 and MYC at 8q24 in chronic lymphocytic leukemia. Clin. Cancer Res. 18, 3791–3802 (2012). 22623730 10.1158/1078-0432.CCR-11-2342 PMC3719990

[b25] Forconi F. . Genome-wide DNA analysis identifies recurrent imbalances predicting outcome in chronic lymphocytic leukaemia with 17p deletion. Br. J. Haematol. 143, 532–536 (2008). 18752589 10.1111/j.1365-2141.2008.07373.x

[b26] Shao H. . Clonally related histiocytic/dendritic cell sarcoma and chronic lymphocytic leukemia/small lymphocytic lymphoma: a study of seven cases. Mod. Pathol. 24, 1421–1432 (2011). 21666687 10.1038/modpathol.2011.102 PMC3175277

[b27] Xie H., Ye M., Feng R. & Graf T. Stepwise reprogramming of B cells into macrophages. Cell 117, 663–676 (2004). 15163413 10.1016/s0092-8674(04)00419-2

[b28] Chen W. . Langerhans cell sarcoma arising from chronic lymphocytic lymphoma/small lymphocytic leukemia: lineage analysis and BRAF V600E mutation study. N. Am. J. Med. Sci. 5, 386–391 (2013). 23923114 10.4103/1947-2714.114172 PMC3731871

[b29] Buser L. . Unique composite hematolymphoid tumor consisting of a pro-T lymphoblastic lymphoma and an indeterminate dendritic cell tumor: evidence for divergent common progenitor cell differentiation. Pathobiology 81, 199–205 (2014). 25228298 10.1159/000365396

[b30] Oh H., Siano B. & Diamond S. Neutrophil isolation protocol. J. Vis. Exp. 17, 745 (2008). 19066523 10.3791/745 PMC3074468

[b31] Cibulskis K. . Sensitive detection of somatic point mutations in impure and heterogeneous cancer samples. Nat. Biotechnol. 31, 213–219 (2013). 23396013 10.1038/nbt.2514 PMC3833702

[b32] Robinson J. T. . Integrative genomics viewer. Nat. Biotechnol. 29, 24–26 (2011). 21221095 10.1038/nbt.1754 PMC3346182

[b33] Carter S., Meyerson M. & Getz G. Accurate estimation of homologue-specific DNA concentration-ratios in cancer samples allows long-range haplotyping. Nat. Preced pp 59–87 (2011).

[b34] Carter S. L. . Absolute quantification of somatic DNA alterations in human cancer. Nat. Biotechnol. 30, 413–421 (2012). 22544022 10.1038/nbt.2203 PMC4383288

[b35] Berger M. F. . The genomic complexity of primary human prostate cancer. Nature 470, 214–220 (2011). 21307934 10.1038/nature09744 PMC3075885

[b36] Chapman M. A. . Initial genome sequencing and analysis of multiple myeloma. Nature 471, 467–472 (2011). 21430775 10.1038/nature09837 PMC3560292

[b37] Fisher S. . A scalable, fully automated process for construction of sequence-ready human exome targeted capture libraries. Genome Biol. 12, R1 (2011). 21205303 10.1186/gb-2011-12-1-r1 PMC3091298

[b38] Li H. & Durbin R. Fast and accurate long-read alignment with Burrows-Wheeler transform. Bioinformatics 26, 589–595 (2010). 20080505 10.1093/bioinformatics/btp698 PMC2828108

[b39] Lohr J. G. . Discovery and prioritization of somatic mutations in diffuse large B-cell lymphoma (DLBCL) by whole-exome sequencing. Proc. Natl Acad. Sci. USA 109, 3879–3884 (2012). 22343534 10.1073/pnas.1121343109 PMC3309757

[b40] McFadden D. G. . Genetic and clonal dissection of murine small cell lung carcinoma progression by genome sequencing. Cell 156, 1298–1311 (2014). 24630729 10.1016/j.cell.2014.02.031 PMC4040459

[b41] Herman S. E. . Ibrutinib-induced lymphocytosis in patients with chronic lymphocytic leukemia: correlative analyses from a phase II study. Leukemia 28, 2188–2196 (2014). 24699307 10.1038/leu.2014.122 PMC4185271

[b42] Qin D., Xia Y. & Whitesides G. M. Soft lithography for micro- and nanoscale patterning. Nat. Protoc. 5, 491–502 (2010). 20203666 10.1038/nprot.2009.234

[b43] Link D. R. . Electric control of droplets in microfluidic devices. Angew. Chem. Int. Ed. Engl. 45, 2556–2560 (2006). 16544359 10.1002/anie.200503540

[b44] Livak K. J. . Methods for qPCR gene expression profiling applied to 1440 lymphoblastoid single cells. Methods 59, 71–79 (2013). 23079396 10.1016/j.ymeth.2012.10.004 PMC3562442

[b45] Marras S., Vargas-Gold D., Tyagi S. & Kramer F. R. Highly selective nucleic acid amplification primers. U. S. Patent Application 20150361475 (2015).

