# Supplementary material for: Clonal evolution in patients with chronic lymphocytic leukaemia developing resistance to BTK inhibition
Source: Nat Commun. 2016 May 20;7:11589. doi: 10.1038/ncomms11589 (PMC4876453; doi:10.1038/ncomms11589)
Supplement: Supplementary Information — Supplementary Figures 1-6 and Supplementary Tables 1-4. [file ncomms11589-s1.pdf]

Supplementary Information

Supplementary Figures

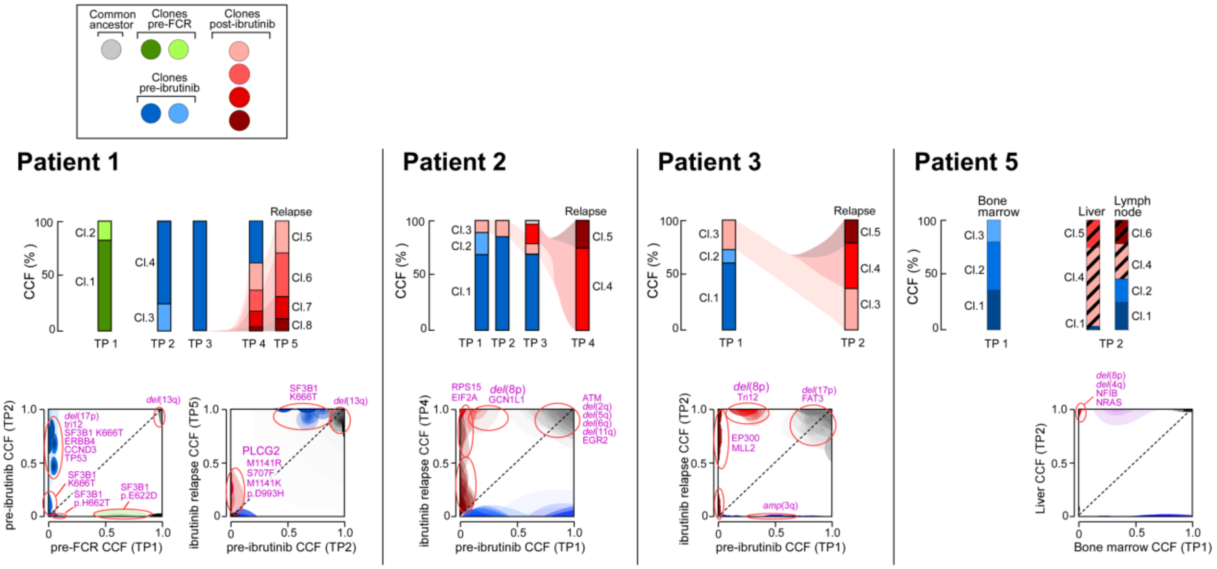

Supplementary Figure 1. Two dimensional clustering enables the distinction of unique clones and the reconstruction of a phylogenetic tree. The phylogenetic relationships were inferred using a serial implementation of 2 dimensional clustering between every two samples in each patient. For each patient, the inferred patterns of clonal evolution are depicted (as in **Figures 1-3**), and representative examples of the 2 dimensional clustering are shown. The individual clones are highlighted with a circle, in addition to candidate driver mutations in each clone.

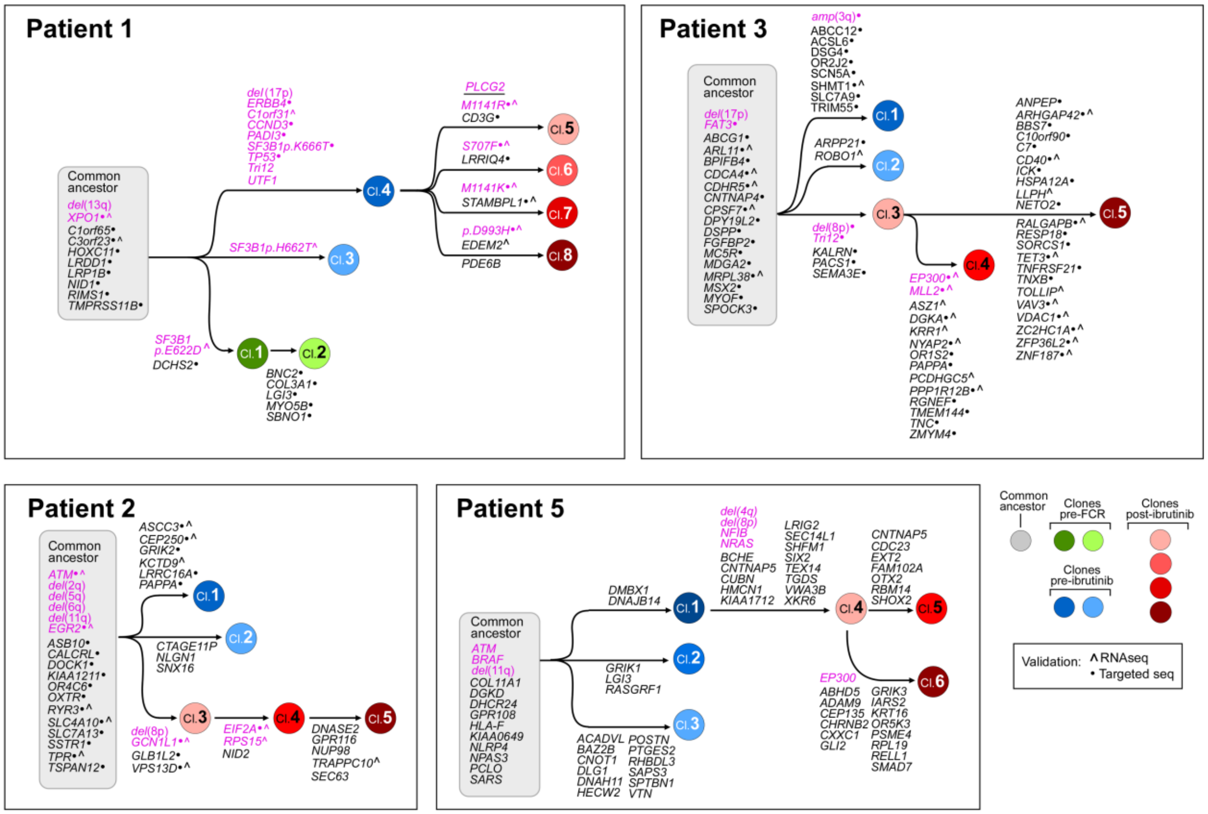

Supplementary Figure 2. Complete mutation annotation overlaid on the phylogenetic tree. For each patient, the mutations are assigned to each clone based on the phylogenetic inference resulting from the serial implementation of 2 dimensional clustering between every 2 samples. Likely candidate drivers are highlighted in pink.

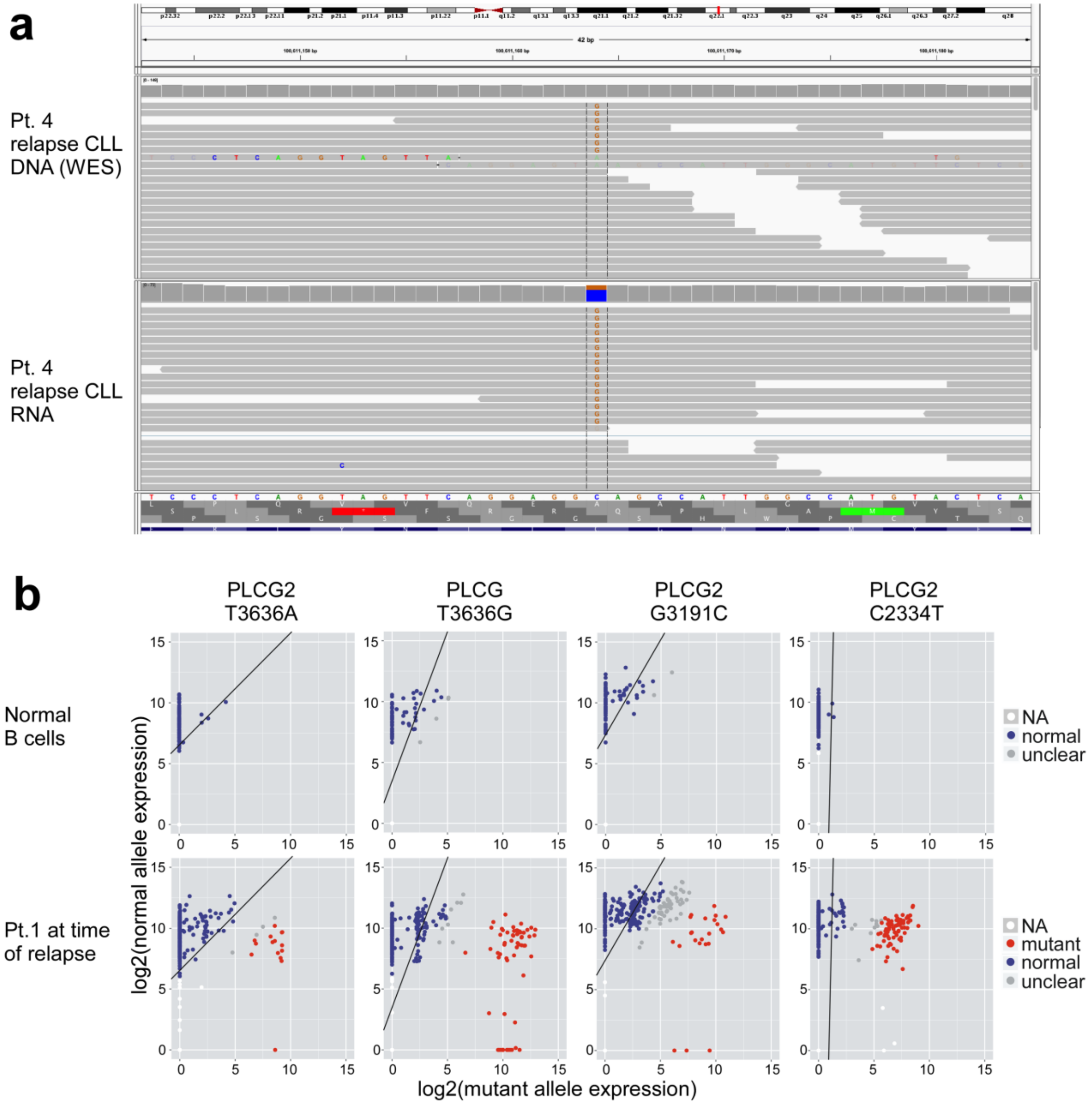

Supplementary Figure 3. A. IGV screenshot of the BTK mutation in Patient 4 CLL cells at the time of relapse. The BTK C481S mutation can be readily detected by both WES of relapsed leukemia cells (top), as well as by matched RNA-sequencing (bottom). Sequence is shown in reverse orientation. These data show a mutation that converts a cysteine at position 481 (TGC) to a serine (TCC). **B**. Single cell expression of wildtype and mutant PLCG2 (at T3636A, T3636G, G3191C, C2334T sites) from cells collected at the of ibrutinib relapse (timepoint (TP) 5) of Patient 1. All data were generated by targeted mutation amplification of single cells on microfluidic chips (Fluidigm Biomark system). Log_2 transformed expression of mutant vs wild-type *PLCG2* alleles are plotted, with each dot representing one single cell. Cells identified to be *PLCG2* wild-type are denoted in blue, while those inferred to be *PLCG2* mutant are red. Cells with ambiguous mutation calls are denoted in grey.

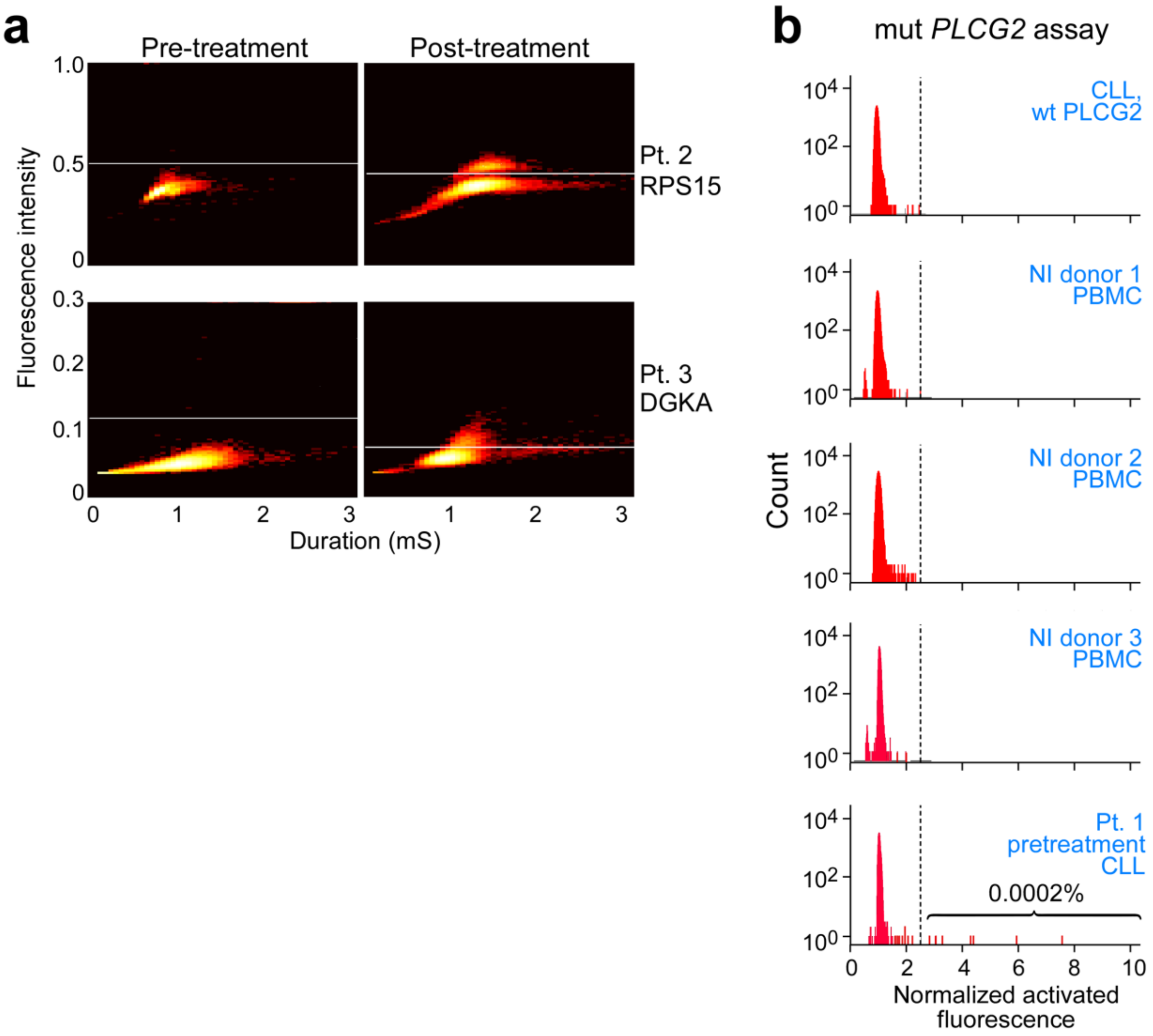

Supplementary Figure 4. **A**. Single cell droplet-PCR detection of resistance cells in Patients 2 and 3 before and after ibrutinib exposure. **B.** Second stage detection of mutated *PLCG2*-specific single cells following digital PCR of merged droplets in 4 negative control samples (a CLL cells with sequence-confirmed PLCG2 wildtype status, and PBMC from 3 normal donors, and Patient 1 pretreatment samples.

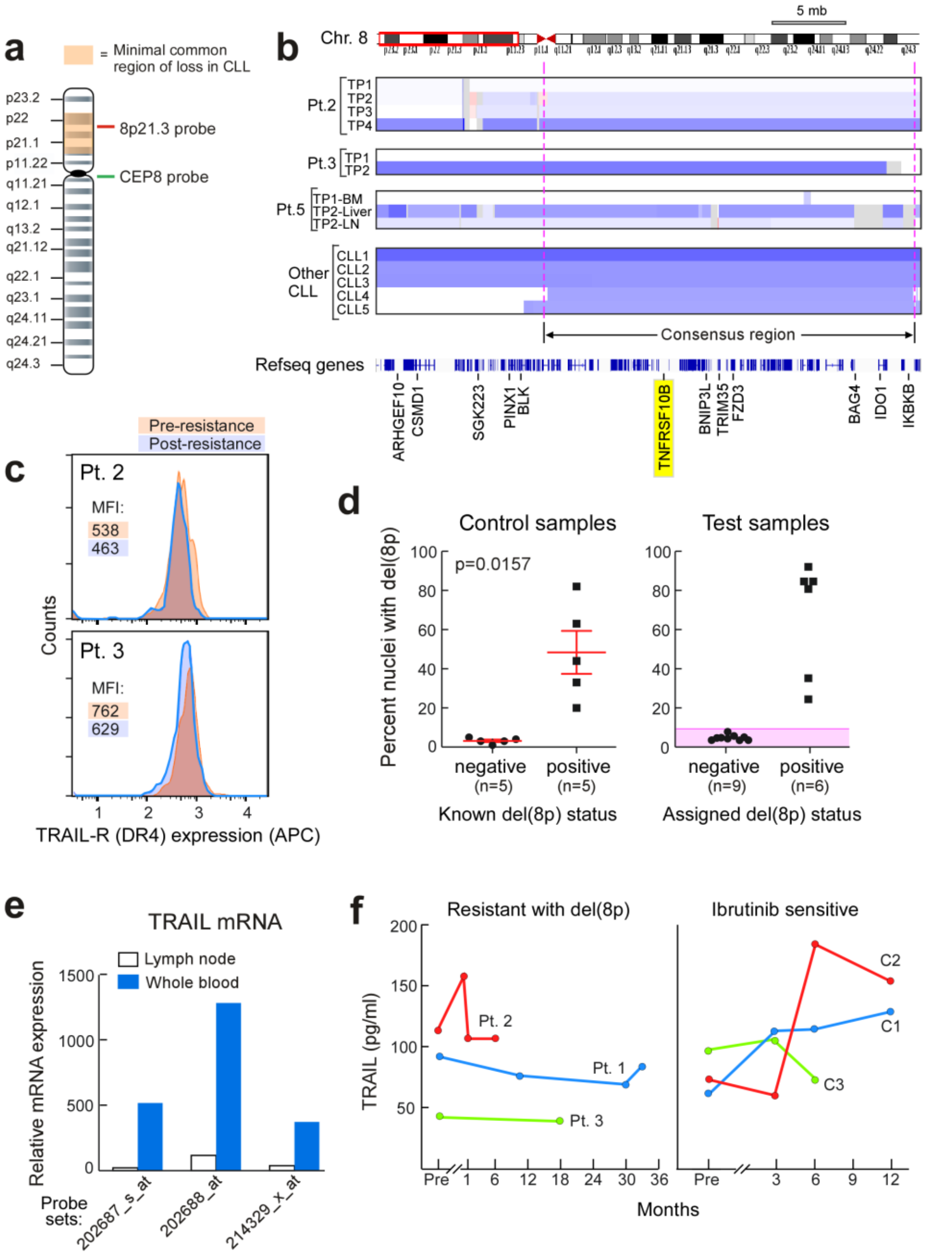

Supplementary Figure 5. Characterization of del(8p) in ibrutinib-resistant CLL patients. A. Schematic of the minimal common region of loss of chromosome 8p in CLLs, to which FISH probes were designed. **B**. SNP array analysis of Patients 2, 3 and 5 and other CLLs from DFCI (CLL1-CLL5) to which deletion in chromosome 8p was detected. **C**. Changes in TRAIL-R (DR4) expression in two CLL patients who developed ibrutinib resistance (Pt 2 and 3) with *del8p*. After gating on CLL cells, lower TRAIL-R expression was noted at time of ibrutinib resistance (blue) compared to prior to treatment initiation (orange). **D**. left- Percentage of nuclei scored with 8p deletion following hybridization to the 8p FISH probe across nuclei from CLL samples, previously characterized by karyotyping as monosomy 8 or deletion of 8p by the BWH Clinical Cytogenetics lab as positive (n=5) or negative (n=5) for deletion in chromosome 8p. For each case, 100 nuclei were scored. The maximum background (i.e. a single 8p21.3 signal) in

negative control specimens was 5%. Based on this data the threshold for considering a sample as positive for del(8p) by FISH is 9.4% (mean of negatives + 3SD). Right- Confirmation of del(8p) status of 9 ‘negative’ and 6 ‘positive’ samples (corresponding to the samples analyzed in **Figure 5C**) through del(8p) FISH of fixed cell pellets. **E**. Visualization of the consistent elevated relative gene expression of TRAIL across probesets in peripheral blood of normal donors compared to lymph nodes, as previously reported [22]. **F**. Comparable levels of TRAIL in plasma of CLL patients with ibrutinib resistance with *del*(8p) (Pts 1, 2, 3) (left panel) or in ibrutinib-sensitive control CLL patients (C1, C2, C3) (right panel). Trended TRAIL levels over time are depicted for each individual patient.

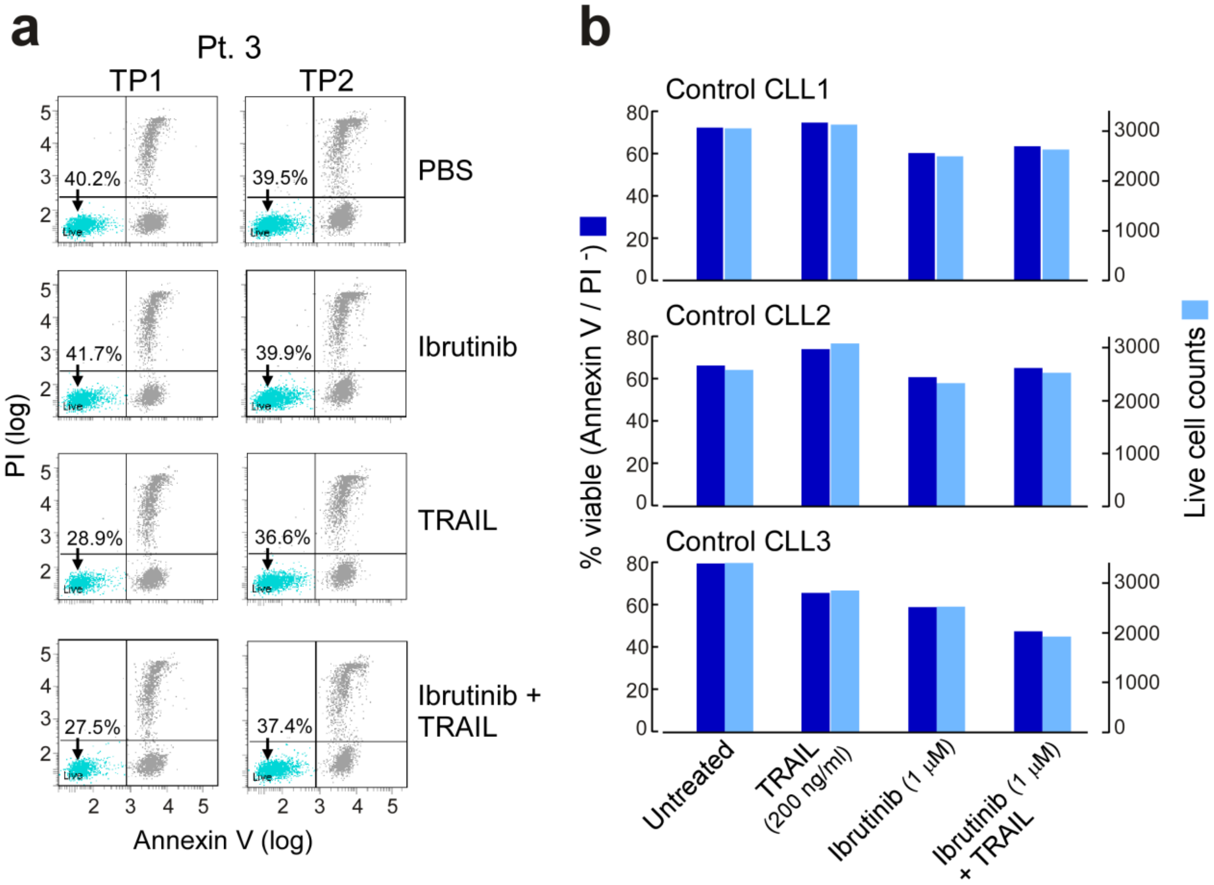

Supplementary Figure 6. A. Cell viability measurements based on flow cytometric analysis following Annexin V and PI of CLL cells from Patient 3 before and after exposure to ibrutinib and/or TRAIL. Live cells constitute the double negative population. **B**. Equivalent levels of % cell viability measured in CLL samples (all without del(8p), confirmed by FISH), whether measured by annexin/PI staining or by live cell counts).

SupplementarySupplementary Tables
Supplementary Table 1: Sequencing coverage and depth Supplementary

Patient_ID	Sequencing_patient_ID	Sample	Mean coverage by WES	Number of unique aligned reads in RNAseq	Median coverage of deep sequencing
**Patient 3**	MDAC-0012	Germline	208.05	NA	2240
**Patient 3**	MDAC-0012	Tumor-TP1	208.16	56112241	1240.5
**Patient 3**	MDAC-0012	Tumor-TP2	208.9	56630057	1727
**Patient 2**	MDAC-0022	Germline	139.19	NA	NA
**Patient 2**	MDAC-0022	Tumor-TP1	74.87	54197779	2286
**Patient 2**	MDAC-0022	Tumor-TP2	134.65	NA	930
**Patient 2**	MDAC-0022	Tumor-TP3	133.58	NA	1264
**Patient 2**	MDAC-0022	Tumor-TP4	112.99	59292360	2832
**Patient 4**	MDAC-0001	Germline	115.6	NA	NA
**Patient 4**	MDAC-0001	Tumor-Bone Marrow-TP1	144.23	NA	NA
**Patient 4**	MDAC-0001	Tumor-Liver-TP2	145.87	NA	NA
**Patient 4**	MDAC-0001	Tumor-Lymph Node-TP2	169.49	NA	NA
**Patient 1**	MDAC-0011	Germline	184.59	NA	1280
**Patient 1**	MDAC-0011	Tumor-TP1	187.98	57911125	5491
**Patient 1**	MDAC-0011	Tumor-TP2	183.76	58399674	3339
**Patient 1**	MDAC-0011	Tumor-TP3	98.44	42811711	1791
**Patient 1**	MDAC-0011	Tumor-TP4	128.62	49050385	4756
**Patient 1**	MDAC-0011	Tumor-TP5	124.31	44876871	2727.5

Supplementary Table 2: **RNAseq normalized expression values. Expression of selected genes by RNAseq at different time points (TP) are displayed, along with the gene names as per Human Genome Organization (HUGO) nomenclature.**

Hugo	Patient #3 TP1	Patient #3 TP2	Patient #2 TP1	Patient #2 TP4
ATP6V1B2	1.42E+01	7.85E+00	9.38E+00	4.94E+00
BIN3	1.00E+01	4.37E+00	9.73E+00	3.58E+00
BNIP3L	1.84E+01	8.24E+00	3.09E+01	1.59E+01
CCDC25	6.01E+00	2.58E+00	7.70E+00	3.31E+00
CNOT7	1.89E+01	7.54E+00	1.48E+01	7.25E+00
CTD-2530N21.4	1.69E+00	9.90E-01	1.00E+00	4.15E-01
CTSB	2.96E+01	1.58E+01	3.06E+01	1.37E+01
DCTN6	5.05E+00	2.89E+00	3.89E+00	2.28E+00
ELP3	7.69E+00	3.79E+00	4.83E+00	1.99E+00
ERICH1	6.45E+00	3.13E+00	5.60E+00	2.18E+00
FAM86B3P	1.25E+00	6.12E-01	1.79E+00	5.43E-01
FDFT1	2.98E+01	1.67E+01	1.86E+01	7.51E+00
FUT10	1.74E+00	5.31E-01	1.48E+00	2.10E-01
GSR	1.50E+01	5.01E+00	1.31E+01	6.26E+00
GTF2E2	1.28E+01	4.96E+00	1.62E+01	5.30E+00
INTS9	7.47E+00	3.72E+00	5.95E+00	2.57E+00
MIR486	2.24E+00	3.26E-01	2.31E+00	5.72E-01
PCM1	9.94E+00	4.27E+00	1.81E+01	7.84E+00
PDLIM2	7.38E+00	2.79E+00	3.14E+00	1.85E+00
PINX1	2.23E+00	8.95E-01	3.13E+00	1.47E+00
PNMA2	1.14E+00	2.02E-01	1.02E+00	1.77E-01
PNOC	3.18E+01	1.52E+01	4.12E+01	1.94E+01
POLR3D	8.57E+00	4.52E+00	6.81E+00	2.17E+00
PPP2R2A	8.29E+00	4.48E+00	6.03E+00	3.14E+00
R3HCC1	7.17E+00	3.30E+00	6.78E+00	3.77E+00
TNFRSF10B	1.43E+01	6.55E+00	2.54E+01	1.41E+01
TNKS	3.12E+00	1.57E+00	2.70E+00	1.31E+00
TRIM35	5.06E+00	2.47E+00	4.43E+00	2.21E+00
VPS37A	2.91E+00	1.09E+00	2.59E+00	1.16E+00
ZDHHC2	1.06E+01	5.35E+00	7.42E+00	4.18E+00

Values in FPKM for genes in the deleted region of short arm of chromosome 8 with a ratio of post/pre <0.6

Supplementary Table 3.
Primers and vectors for the cloning of PLCG2 and RPS15 minigenes

Gene	Mutation Site	Primers (5’-3’)	Vector	Selection Antibiotic	Dose
PLCG2	M1141R	F: GCACTCAATTTCCAGACGGC<br>R: GAAAACCAGGAGGGAAGCCA	pcDNA3.1/ V5-His	Neomycin	1mg/ml
RPS15	G129V	F: CCACCATGGGAAGCCGGAAGTGGTGAAGA<br>R: TTAGTCGACGCGCCTTTATTAGCTGAGCC	pEFpuro	Puromycin	5μg/ml

Supplementary Table 4.

For single cell Fluidigm/Biomark detection of the mutated variants of PLCG2 and SF3B1, single cell cDNA were first preamplified using the primers in sTable 5A, and then amplified for targeted detection of the mutated vs wildtype allele by RT-PCR (sTable 5B). Single cell droplet PCR detection of mutated alleles used the primers in sTable 5C. Some primers were used in common between the procedures (shaded in grey).

A. Primers for preamplification of single cell cDNA

Gene	***RefSeq***	***Mutation***		***Preamplification primers (5'→ 3')***	
				Forward	***Reverse***
SF3B1	NM_012433	A2045C		cgaCCCTTCTTAAAAGCTGTGTGCAA	cgaTCTAAGATGTGGCAAGATGGCA
SF3B1	NM_012433	A2033T		cgaCCCTTCTTAAAAGCTGTGTGCAA	cgaTCTAAGATGTGGCAAGATGGCA
SF3B1	NM_012433	G1914T		tcgGGAAGGCCGAGAGATCATTTCTAA	cgaGCAGAGGCTACAACAGCAA
PLCG2	NM_002661	T3636A	M1141K	cgtACACAGGAGAAGGTGACATTTGAA	cgaCTTCAGAGGAACGGACCTGAA
PLCG2	NM_002661	T3636G	M1141R	cgtACACAGGAGAAGGTGACATTTGAA	cgaCTTCAGAGGAACGGACCTGAA
PLCG2	NM_002661	G3191C	D993H	cgaCGTCGACCTCCTGAAGTACAA	cgaTGAGTGCCACCATCTGAGAA
PLCG2	NM_002661	C2334T	S707F	cgaCAAGGTAAAGCATTGTCGCATCAA	cgaCGGTAGAGTGAATGCTTCTCGTA
RPS15	NM_001018	G448T		tcgCCGAGATGATCGGCCACTA	cgaGAGCCATTACTTGAGAGGGATGAA
DGKA	NM_001345	G2415A		acgTGCAGACGCCCTGTACAA	cgaCCCCTTAGCTCAAGAAGCCAA

B. Primers for targeted single cell detection of wildtype vs normal alleles with Fluidigm/Biomark

Gene	***RefSeq***	***Mutation***		***qPCR primers (5'→ 3')***		
				Normal	***Mutant***	***Locus-specific***
SF3B1	NM_012433	A2045C		CAAGAAGTCCTGGCAAGCGAGtatatccatactGTATTAA	CAAGAAGTCCTGGCAAGCGAGtatatccatactGTATTAC	cgaGGCACAGCCCATAAGAATAGCTA
SF3B1	NM_012433	A2033T		AGCCCATAAGAATAGCTATCTGTTGTACAATCacttctttctaCAGTGTG	AGCCCATAAGAATAGCTATCTGTTGTACAATCacttctttctaCAGTGAG	cgaCAAAAGCAAGAAGTCCTGGCAA
SF3B1	NM_012433	G1914T		ACAACAGCAAAAGCTCTAGCTGTTGaccatacaactaGGACATACT	ACAACAGCAAAAGCTCTAGCTGTTGaccatacaactaGGACATAAT	cgaGCTGCTGGTCTGGCTACTA
PLCG2	NM_002661	T3636A	M1141K	GGGTAAGTGGCATGAGCAAGAAAGacctctaatCTGAACAT	GGGTAAGTGGCATGAGCAAGAAAGacctctaatCTGAACTT	cgtTTTCTGCGCTTTGTGGTTTATGAA
PLCG2	NM_002661	T3636G	M1141R	GGGTAAGTGGCATGAGCAAGAAAGacctctaatCTGAACAT	GGGTAAGTGGCATGAGCAAGAAAGacctctaatCTGAACCT	cgtTTTCTGCGCTTTGTGGTTTATGAA
PLCG2	NM_002661	G3191C	D993H	CACAGCCAGAGGCGGAAGtctaacGAAGAGTCA	CACAGCCAGAGGCGGAAGtctaacGAAGAGTG	cgaGCGTCTACCCAAAGGGACAA
PLCG2	NM_002661	C2334T	S707F	GACGGCCGGCACTTTGTtactctcaacctGACCTCC	GACGGCCGGCACTTTGTtactctcaacctGACCTTC	cgaGAGCTCCACCAGACTCTCAA

RPS15	NM_001018	G448T		AGTGGGTGGCCCCGATtaatcatacaactCCGGC	AGTGGGTGGCCCCGATtaatcatacaactCCGGA	cgaCCATCACCTACAAGCCCGTAA
DGKA	NM_001345	G2415A		CCAAAGAAATTGGTGGAGCGGGtctttCATGGGCA	CCAAAGAAATTGGTGGAGCGGGtctttCATGGGTA	acgTCAAGATCACCCACAAGAACCA

C. Primers for qPCR detection of mutated alleles using single cell droplet PCR

Gene	***RefSeq***	***Mutation***		***qPCR primers (5'→ 3')***		
				Locus-specific primer	***Mutant***	***Probe***
PLCG2	NM_002661	T3636A	M1141K	cgtACACAGGAGAAGGTGACATTTGAA	GGGTAAGTGGCATGAGCAAGAAAGacctctaatCTGAACTT	6FAM-CCACAAAGCGCAGAAATGCC-MGBNFQ
RPS15	NM_001018	G448T		tcgCCGAGATGATCGGCCACTA	AGTGGGTGGCCCCGATtaatcatacaactCCGGA	6FAM-ACGGGCTTGTAGGTGATGGA-MGBNFQ
DGKA	NM_001345	G2415A		acgTGCAGACGCCCTGTACAA	CCAAAGAAATTGGTGGAGCGGGtctttCATGGGTA	NED-CTGGTTCTTGTGGGTGATCTTG-MGBNFQ
